# Biodiversity of New Lytic Bacteriophages Infecting *Shigella* spp. in Freshwater Environment

**DOI:** 10.3389/fmicb.2021.619323

**Published:** 2021-02-17

**Authors:** Khashayar Shahin, Mohadeseh Barazandeh, Lili Zhang, Abolghasem Hedayatkhah, Tao He, Hongduo Bao, Mojtaba Mansoorianfar, Maoda Pang, Heye Wang, Ruicheng Wei, Ran Wang

**Affiliations:** ^1^Key Lab of Food Quality and Safety of Jiangsu Province-State Key Laboratory Breeding Base, Jiangsu Academy of Agricultural Sciences, Institute of Food Safety and Nutrition, Nanjing, China; ^2^Curtin Medical School, Curtin University, Perth, WA, Australia; ^3^Suzhou Institute of Nano-Tech and Nano-Bionics, Chinese Academy of Sciences, Suzhou, China

**Keywords:** water, phage, biodiversity, bacteriophage, *Shigella*, *Enterobacteriaceae*

## Abstract

Bacteriophages, viruses that infect and replicate within prokaryotic cells are the most abundant life forms in the environment, yet the vast majority of them have not been properly reported or even discovered. Almost all reported bacteriophages infecting the *Enterobacteriaceae* family, with *Escherichia coli* being the major subject of studies, have been isolated from wastewater, sewage, and effluent resources. In the present study, we focused on the distribution and biodiversity of *Shigella* phages in an aquatic ecosystem. While no *Shigella* bacteria was recovered from the Yangtze River, three lytic phages were isolated from this ecosystem and were subjected to biological, morphological, and genomic characteristics. Comparative genomics and phylogenetic analyses demonstrated that vB _SflM_004 isolate belongs to *Myoviridae* family, *Felixounavirus* genus of *Ounavirinae* subfamily, vB_SdyM_006 was classified under the same family, however, it is suggested to be in a new genus under *Tevenvirinae* subfamily with some other related bacteriophages. vB_SsoS_008 phage belongs to the *Siphoviridae* family, *Tunavirus* genus, *Tunavirinae* subfamily. The phages did not harbor any genes involved in the lysogenic cycles and showed a high temperature and pH stability. The biodiversity of the isolated phages highly suggests that continued isolation on non-model members of *Enterobacteriaceae* family is necessary to fully understand bacteriophage diversity in aquatic environments.

## Introduction

Bacteria-infecting viruses or bacteriophages (phages) are the most abundant biological entities on planet earth (Suttle, 2005). With an estimated minimum number of 10^31^ bacteriophages have the highest diversity with respect to genetics, morphology, host range, and infection cycles (Hendrix et al., 2002). Our knowledge on bacteriophages is extremely limited for three main reasons. First, in theory, each and every bacterial species is a host for at least a phage. With our current knowledge on the field and the state of the art and technology, only a small percentage of bacteria can be grown *in vitro* thus the majority of the microorganisms are uncultivated (or yet-to-be-cultivated; Hatfull and Hendrix, 2011). Second, of the cultured one, only very few numbers have been used as hosts for phage isolation (Hatfull and Hendrix, 2011). Finally, even with this limited number of host cells, almost all of the phages reported so far have been isolated from environments such as untreated sewage and hospital wastewater or wound since there is a higher chance of isolating bacteriophages using such resources due to these resources being the most contaminated ecosystem containing a high number of pathogens. Considering the above facts, isolation of phages from other aquatic environments such as freshwater resources and reservoirs like rivers and lakes is of particular importance in view of their impact on both the microbial diversity and the ecological fate of photogenic bacteria.

*Shigella* is a gram-negative bacterial genus including four species: *Shigella boydii*, *Shigella dysenteriae*, *Shigella flexneri*, and *Shigella sonnei* (Shahin et al., 2019c), all causing shigellosis with hundreds of millions of food/water-borne infections annually (WHO, 2005). Although shigellosis can be usually treated with antibiotics, propagation of antibiotic-resistant strains has created many serious health problems in recent years (Baker et al., 2018), for example over the past few decades several shigellosis outbreaks have been reported in the geographical regions (Löfdahl et al., 2009; He et al., 2012; Abaidani et al., 2015; Puzari et al., 2018).

As an alternative to antibiotic, lytic bacteriophages can be used to control or to treat bacterial infections, a process is known as phage therapy (Shahin et al., 2019d; Bao et al., 2020). Despite the worldwide distribution of *Shigella* species, the high number of infected cases, and the great importance of food safety, only very few *Shigella*-infecting phages have been identified, studied, and reported so far. Almost all of these reported phages have been isolated from raw sewage samples. *Myoviridae* phages (pSs-1 and WZ1; Jamal et al., 2015; Jun et al., 2016a), *Siphoviridae* phages (vB_SflS-ISF001, vB_SsoS-ISF002, vB-SdyS-ISF003, SH6, Shfl1, and pSf-2; Jun et al., 2013, 2016b; Hamdi et al., 2017; Shahin et al., 2018, 2019a,b), and *Podoviridae* phages (pSb-1 and Sf6; Casjens et al., 2004; Jun et al., 2014) are among the phages that infect different species of *Shigella*. Moreover, although phage therapy for controlling *Shigella* has a long history as the first phage research was done by Felix d’Herelle in the 1910s (Duckworth, 1976), nevertheless there is no comprehensive research on the abundance, distribution, and diversity of *Shigella* in natural aquatic environments yet.

Hence, in this study, we focused on isolation and subsequent morphological, biological and genomic characterization of *Shigella*-infecting phages from Yangtze River as one of the biggest freshwater resources on the planet earth.

## Materials and Methods

### Bacterial Stains and Growth Condition

All the *Shigella* bacteria used in the present study (Supplementary Table S1) have been previously isolated. They were all stored in tryptic soy broth (TSB; Merck, Germany) containing 30% glycerol and kept at −70°C in the central bacterial strains collection of International Phage Research Center (IPRC) containing 30% glycerol at 70°C. In addition to these isolates, the type-stains of *S. sonnei* (ATCC 9290), *S. flexneri* (ATCC 12022), *S. dysenteriae* (PTCC 1188), *S. boydii* (ATCC 9207), and *Escherichia coli* (ATCC 25922) were used for the determination of the bacteriophages host range. All the isolates were cultured routinely on brain heart infusion (BHI) agar (Merck, Germany) or in BHI broth (Merck, Germany) with constant shaking at 200rpm and 37°C.

### Bacteriophages Isolation and Morphology Analysis

Four *Shigella* isolates including *S. flexneri* (w7) and *S. sonnei* (w44) which had been isolated earlier from other freshwater sources and also showed the highest antibiotic resistance, as well as *S. dysenteriae* (s.d.f1) and reference strain *S. boydii* (ATCC 9207), were used individually as the host bacteria for phages isolation following the previously described method with a slight modification (Yazdi et al., 2020). Briefly, 100ml of Yangtze River water samples [GPS coordinates of sampling locations include: (1) latitude: 32°06′37.8″N and longitude: 118°44′56.1″E; (2) latitude: 32°09′25.9″N and longitude: 118°50′48.8″E; Supplementary Figure S1] was centrifuged (10min at 6,000 × g) and filtrated through 0.45μm sterile syringe filters (JinTeng, China). The filtrate was used for phage isolation with pre-enrichment (method I) or without pre-enrichment (method II) pre-enrichment. In method I, 50ml of the filtrate water was added to 50ml of the early-exponential culture of the host bacteria and incubated overnight with constant shaking (200rpm). After centrifugation (10min, 8,000 × g) and filtration (0.45-μm), 20 μl of the filtrate was spotted on BHI plates overlaid with the individual strain. In method II, 20 μl of the filtrate water sample was dropped directly onto the surface of lawn cultures of the host bacteria and incubated at 37°C for at least 24h. Emergence of clear plaques either in method I or method II were considered primary as the presence of a lytic phage. Then a single plaque was picked up carefully for phage purification procedure using three repeats of single plaque isolation, elution, and re-plating. Phage propagation and phage titers determination were carried out regularly according to Clokie and Kropinski’s (2009) protocols.

### Phages Transmission Electron Microscopy

The phage lysates (10^9^PFU ml^−1^) were precipitated using NaCl (0.5M) and PEG 8000 (10%, Amresco, Solon, United States) and then highly purified using centrifugation on cesium chloride gradient as described by Clokie and Kropinski (2009). The phages were negatively stained with 2% phosphotungstic acid (PTA) for 1min and left to dry for 30min and finally, the grids were analyzed in a Hitachi HT7700 transmission electron microscope at an operating voltage of 100kV at Nanjing Agricultural University (NAU), Nanjing, China.

### Host Range

The host ranges of the phages were determined using spot assays (Shahin and Bouzari, 2018). Ten microliters of the phage suspension (10^9^PFU ml^−1^) was spotted onto double-layered BHI agar plates of each host strains (Supplementary Table S1). After an overnight incubation, the plates were checked for the appearance of clear plaque (++), turbid plaque (+), or no plaque (−). The efficiency of plating assay (EOP) was carried out against the phages-sensitive isolates of *Shigella* spp. as described previously (Shahin et al., 2020). The EOP of the phage on their primary host strain (reference host) was considered as 1 and EOP of each strain were calculated as the ratio of phage titer on the tested bacterium to the phage titer on the reference host.

### The Phages Biological Characterizations

*Shigella flexneri* (w7), *S. dysenteriae* (s.d.f1), and *S. sonnei* (w44) were used as host bacteria in all the experiments. The thermostability and pH stability of the phages were evaluated by incubation of the phages lysate (10^9^PFU ml^−1^) at wide range of temperatures (−20 to 80°C) and pH (2–12) as previously described (Shahin et al., 2018). Briefly, 1ml of phage solution (10^9^pfu/ml) was incubated at −20 to 70°C individually for 1h to evaluate the bacteriophage stability to different temperatures. Moreover, to measure the tolerance of isolated phages to different pH values, they were added to microtubes containing sterile SM buffer at pH values of 3−12 and then incubated for 1h. The survival phages in both examinations were measured using the overlay method and the titer is reported as a percentage of the control sample titers. The phages adsorption rates to the surface of their bacterial hosts were determined as previously described (Kropinski et al., 2009). Moreover, one-step growth experiments were done to determine phages burst size and latent periods (Shahin et al., 2018). The bacteriolytic potential of the phages were evaluated by monitoring the changes in OD_600_ absorbance of the phage/host mixture at different MOI as described previously (Shahin and Bouzari, 2018). The assays were performed in triplicate.

### DNA Extraction, DNA Fingerprinting, and Whole Genome Sequencing

The stocks of purified phages were condensed using ultracentrifugation at 105,000 × g for 3h at 4°C (Beckman Optima L-80 XP ultracentrifuge, TYPE 45 Ti rotor). The pellet was diluted in SM buffer and treated by 10μg/ml DNase I and RNase I (Sigma, China) to digest any free DNA and RNA. The genomic DNA of phages was then extracted using phenol/chloroform/isoamyl alcohol protocol as described previously by Sambrook and Russell (2001). Finally, the quality and quantity of the extracted DNA were examined using agarose gel electrophoresis and NanoDrop (Thermo Scientific, United States).

The digestion patterns produced by *EcoRI*, *EcoRV*, and *HindIII* restriction enzymes (Thermo Fisher Scientific, United States) were used for DNA fingerprinting analysis. The phage DNA and the endonuclease were mixed individually according to the manufacturer’s protocol. After the incubation period, the DNA fragments were separated by 1% agarose gel electrophoresis at 90V for 60min. The DNA libraries and whole-genome sequences were obtained using Illumina HiSeq NGS DNA sequencing system (TGS, Shenzhen, China). The raw sequencing data were assembled using SOAPdenovo (v2.04) at the default setting and the assembled sequences were deposited at DDBJ/EMBL/GenBank (Table 1).

**Table 1:** Morphologic and genomic characteristics of the isolated phages in current study.

	vB_SflM_004	vB_SdyM_006	vB_SsoS_008
Host	*Shigella flexneri*	*Shigella dysenteriae*	*Shigella sonnei*
Isolation method	II	I	I
Plaque size	1.5–2mm	2mm	2.5–2.7mm
Head diameter	99.6 ± 8nm	92.7 ± 2nm	59.2 ± 2nm
Tail length (relaxed form)	107.2 ± 6nm	106.2 ± 4	171.9 ± 5
Width (relaxed form)	15.5 ± 1nm	15.5nm	6.9 ± 1nm
Tail length (contracted form)	50.2 ± 2nm	54.1 ± 1nm	–
Width (contracted form)	15.8 ± 1nm	15 ± 1nm	–
Family	*Myoviridae*	*Myoviridae*	*Siphoviridae*
Sequencing platform	Illumina HiSeq	Illumina HiSeq	Illumina HiSeq
Fold coverage	4,115×	3,247×	1,405×
Genome length	85,887bp	166,138bp	50,414bp
G+C%	38.6	31.5	45.6
No. of coding sequences	135	252	83
tRNA	0	9	0
GenBank ID	MK295205	MK295204	MK335533

### Bioinformatic Analysis

Open reading frames (ORFs) were detected using Prokaryotic GeneMark.hmm version 3.25^1^ (Besemer et al., 2001) and NCBI ORF Finder^2^ and translated to protein sequences using ExPASy translate tool.^3^ Molecular weight and isoelectric pH of the predicted ORF were estimated using ExPASy compute pI/Mw tool^4^ (Gasteiger et al., 2005). tRNAscan-SE was used to find any tRNA sequence (Schattner et al., 2005). Functional and conserved domains of the predicted ORFs proteins were analyzed using a couple of software and online tools including Basic Local Alignment Search Tool (BLASTp),^5^ HHpred^6^ (Zimmermann et al., 2018), Pfam^7^ (Finn et al., 2015), and InterProScan^8^ (Altschul et al., 1997). The promoter sequences were identified using BPROM program of the Softberry website with the maximum allowable distance from the starting codon of a gene at 100bp (Salamov and Solovyevand, 2011).

### Comparative Genomic Analysis and Phylogeny

The whole-genome sequences of the taxonomically close and related phages were obtained from NCBI database^9^ and were used for comparison of the sequences at both genome and proteome levels using EasyFig v 2.2.3 (Sullivan et al., 2011). In addition, CoreGenes 3.5 was used for core gene analysis (Zafar et al., 2002). The phylogenetic tree was constructed using Mega 7.0 based on unweighted pair group method with arithmetic mean (UPGMA) with 2,000 bootstrap replication (Kumar et al., 2016).

## Results

### Phages Isolation and Morphology

**Figure 1:**
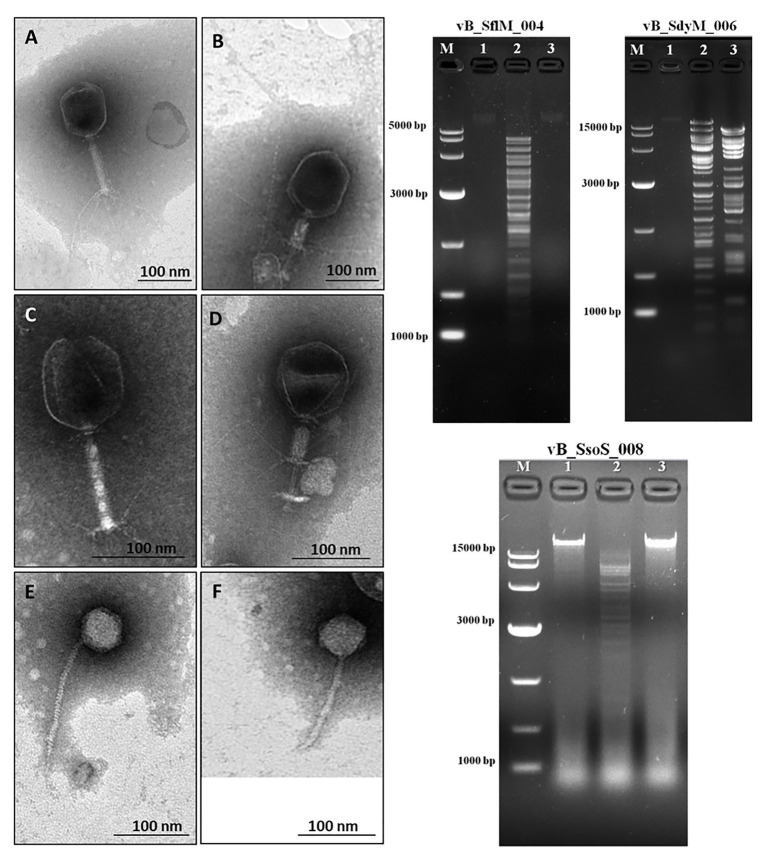
(Left side) The electron micrograph of phages vB _SflM_004 **(A,B)**, vB_SdyM_006 **(C,D)**, and vB_SsoS_008 **(E,F)**. The samples were negatively stained with 2% phosphotungstic acid (PTA). Scale bars 100nm. (Right side) The DNA fingerprinting analysis of the genomic DNA of phage vB _SflM_004, vB_SdyM_006, and vB_SsoS_008. The genome was digested with *EcoRI* (line1), *EcoRV* (line2), and *HindIII* (line3). M line represents the DNA marker.

Phages were isolated from Yangtze River according to their ability to lyse and generate clear plaques with or without hallow zones using several *Shigella* species as the host cells. The transmission electron microscopy micrographs show that the isolated phages for *S. flexneri* and *S. dysenteriae* have an icosahedral head, contractile tail, collar, and base plate, the typical properties *Myoviridae* family of bacteriophages (Figures 1A-D). Moreover, the TEM micrograph of the isolated phage for *S. sonnei* shows that it has an icosahedral head and a non-contractile tail, a similar structure to that of the *Siphoviridae* phages (Figures 1E,F). The head diameter, tail length, and width of the phages are summarized in Table 1. The phages were designated as vB_SflM_004, vB_SdyM_006, and vB_SsoS_008 according to their host species and phage family.

### Bacteriophages Host Ranges

The host range of the isolated phages were tested on a wide range of bacteria including *Shigella* isolates, as well as standard strains of gram-negative and gram-positive bacteria. The vB_SdyM_006 phage was capable of producing clear plaque only on *S. dysenteriae* isolates (3/3 isolates), while vB_SflM_004 and vB_SsoS_008 produced either clear or cloudy plaques on most of the tested *S. flexneri* and *S. sonnei* isolates (Supplementary Table S1). A relatively wide range of EOP (0.12 ± 0.07 ~ 1) of the phages was observed against different isolates of *Shigella* spp. (Supplementary Table S2).

### DNA Fingerprinting

The DNA fingerprinting of the isolated phages were obtained using restriction endonucleases *EcoRI*, *EcoRV*, and *HindIII*. The obtained restriction pattern revealed that the genome of the phages vB _SflM_004 and vB_SsoS_008 were digested only with *EcoRV* and the genome of phage vB_SdyM_006 was digested with *EcoRV* and *HindIII*. The observed differences in the DNA fingerprints in terms of size and pattern (Figure 1) imply that the genome size and sequence of the isolated phages were different from each other.

### Basic Biological Characteristics

**Figure 2:**
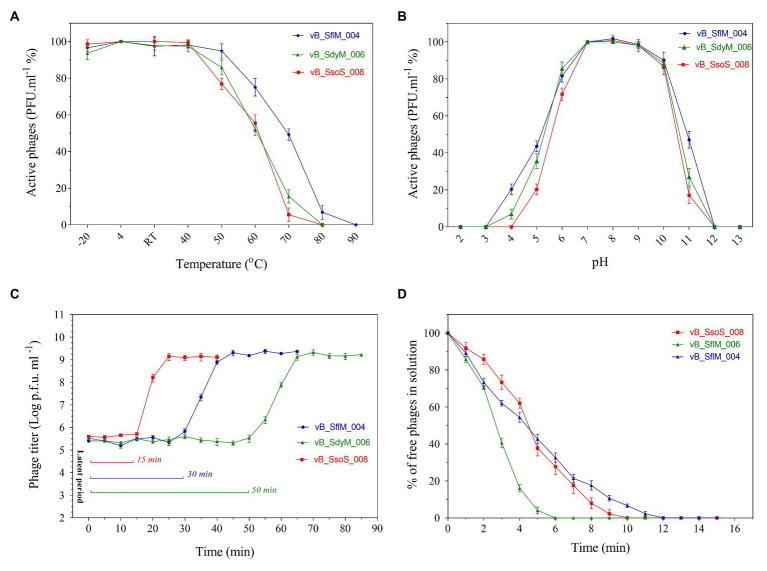
The thermostability **(A)**, pH stability **(B)**, one-step growth curve **(C),** and adsorption rate **(D)** of vB _SflM_004, vB_SdyM_006, and vB_SsoS_008. The error bars indicate standard deviations (SD).

The thermo‐ and pH-stability of the phages were tested at a wide range of temperatures and pH values (Figure 2). Titer of all the three phages were stable (>90%) at −20 to 40°C, but it started to decrease when incubated at 50°C for 1h. While by further increase in the temperature to 80°C, vB_SdyM_006 and vB_SsoS_008 could not be recovered, the vB _SflM_004 phage was still recovered at this temperature, but lost its activity when incubated at 90°C (Figure 2A). In the case of pH stability, the highest activity was observed at pHs ranging from 6 to 8. Incubation at basic pH of 12 (for all phages) and acidic pHs of 4 (for vB_SsoS_008) and 3 (for vB_SdyM_006 and vB _SflM_004) led to deactivation of the phages (Figure 2B). The one-step growth curves demonstrated that the phages vB _SflM_004, vB_SdyM_006, and vB_SsoS_008 were started to release from their host cells after 30, 50, and 15min, respectively. Moreover, the burst sizes were estimated to be about 139 ± 29, 93 ± 15, and 94 ± 9 virions per single bacterium for vB _SflM_004, vB_SdyM_006, and vB_SsoS_008, respectively (Figure 2C). Additionally, as shown in Figure 2D the phages particles were absorbed immediately after incubation where vB _SflM_004, vB_SdyM_006, and vB_SsoS_008 phages were fully absorbed on their host cell after 12, 6, and 10min, respectively.

### Genome Analysis

The fold coverage, genome size, G+C contents, and other general genome features of the phages vB_SflM_004, vB_SdyM_006, and vB_SsoS_008 are presented in Table 1.

#### Genome Analysis of vB_SflM_004

**Figure 3:**
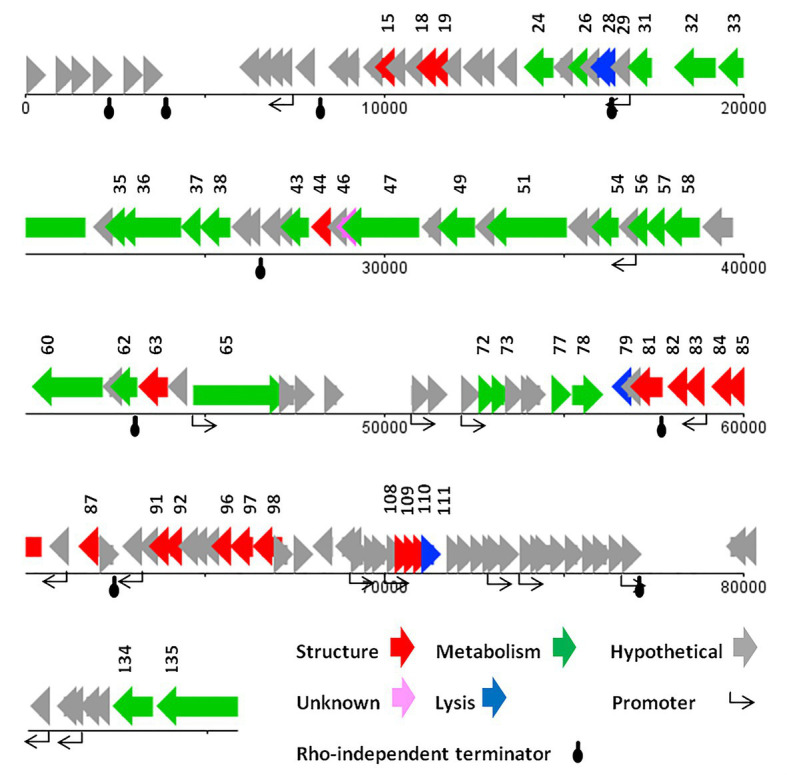
The linear genome map of phage vB_SflM_004.

ATG was detected as the start codon in all of the ORFs. The Opal (TGA), Ochre (TAA), and Amber (TAG) stop codons were presented in 59, 49, and 20 ORFs, respectively. The BPROM search detected 16 promoters (Supplementary Data 1) with consensus sequences at −10 (tttTAtaaT) and −35 (TTcAca; the capital letters indicated conserved nucleotides). Nine Rho-factor independent termination sites were also detected in the vB_SflM_004 genome with FindTerm online software (Figure 3).

In general, the genomic organization and genetic analysis of the phage vB_SflM_004 demonstrated that the genome contained 135 possible ORFs including 20 ORFs encoding structural proteins, 25 ORFs for metabolism-related proteins, four ORFs associated with bacterial lysis-like proteins, 83 ORFs encoding hypothetical proteins which showed relatively high similarity to the previously described phage hypothetical proteins with no clear understanding of their functions yet, and the three remaining ORFs which showed no similarity to any known proteins in the databases (Figure 3). The list of 135 ORFs, as well as their details and annotation, is provided in Supplementary Data 2. The detected genes involved in the bacterial cell lysis were lysozyme (*gp111*), holin (*gp79*), and two spanin (o-spanin, *gp28* and i-spanin, *gp29*) which are similar to the previously reported genes in the *Ounavirinae* subfamily including *Felixounavirus* (*gp28* and *gp29*), *Mooglevirus* (*gp111*), and *Suspvirus* (*gp79*). In addition, two pairs of rIIA/rIIB proteins were also detected at the semi-beginning (*ORF32* and *ORF33*) and the end (*ORF134* and *ORF135*) of vB_SflM_004 genome which could play role in regulation of bacterial lysis (Supplementary Data 2). The gene products involved in the metabolism/regulation pathways of vB_SflM_004 were identified as different types of DNA polymerases, kinases, reductases, protease, nucleases, hydrolysis, and regulatory proteins with relatively high similarity to those of *Ounavirinae* subfamily (check Supplementary Data 2 for more detail). The structural and assembly genes were encoding the tail fiber proteins, tail sheath, tail protein, tail tube protein, minor tail protein, tail assembly protein, major capsid, pro-head assembly scaffold protein, and a head maturation protease. Some of these proteins were similar to those available in the GenBank database. For instance, tail tube, major capsid, and tail protein were almost identical (≥97%) to the respective predicted gene products of phages vB_EcoM_Alf5, SF19, Meda, and SF13. On the other hand, the tail proteins and the major capsid protein represented a low identity (≤55%) to the previously reported phage proteins. The gene distribution pattern (Figure 3 and Supplementary Data 2) shows that about half of the gene products of ORF81 to ORF110 were identified as structural proteins. Same as other viruses, bacteriophages tend to have the genes with similar function close to each other in a compact arrangement (Bardina et al., 2016). Thus, it is possible that the remaining ORFs (which have been considered as hypothetical proteins) in this region of the genome may have a structural function.

The highest similarity of the hypothetical proteins was to those of phage SF13 (13 out of 83 ORF) with a clear concordant relation in their gene products function and their respective identified conserved domains. However, in the case of conserved domains of DUF3277 and DUF3383 no clear relations were found (Supplementary Data 2).

**Figure 4:**
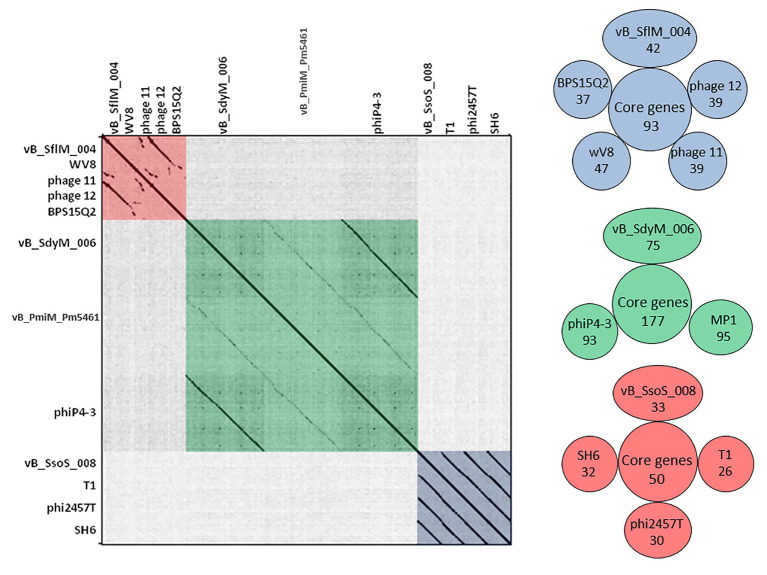
(Left side) Dot plot alignment of the nucleotide sequences of the isolated phages vB _SflM_004, vB_SdyM_006, and vB_SsoS_008 with that of the close related phages. The FASTA file of 1,300,800bp file was compared to itself using GEPARD. Dark diagonal lines parallel to the main diagonal shows strong and continuous sequence similarity, while pale lines indicate weaker sequence relationships due to interruptions that result in a discontinuous line. The red, green, and blue boxes were added to illustrate the phage sequences related to vB _SflM_004, vB_SdyM_006, and vB_SsoS_008. (Right side) The shared conserved proteins of vB _SflM_004, vB_SdyM_006, and vB_SsoS_008 phages with their close related phages (SH6, Shfl1, ADB-2, JMPW2). Only the gene products with >75 score in CoreGenes analysis were considered as a homolog.

BLASTN analysis of the phage vB_SflM_004 genome revealed that the genome of the phage was highly similar (~94% similarity with >75% query coverage) to *E. coli* phage 11, phage 12, *Enterobacteria* phage WV8, and *Salmonella* phage BPS15Q2. As shown in (Figure 4) the dot plot analysis of these bacteriophages using Gepard demonstrated a considerable sequence similarity between vB_SflM_004 and the other related phages with a few remarkable differences such as deletion of an ~10kb region at position around 18,000. Comparison of the genome with other close phages using CoreGenes showed that 68% of the proteins were shared with *Ounavirinae* subfamily in which the entire lysis group proteins, some genes with structural or regulatory functions, as well as some of the hypothetical proteins were conserved (Figure 4 and Supplementary Data 3). Supplementary Table S4 summarized the comparison of the basic genomic properties and Supplementary Figure S2 depicts the relatedness of vB_SflM_004 and other phages with high homology using Easyfig software.

#### Genome Analysis of vB_SdyM_006

**Figure 5:**
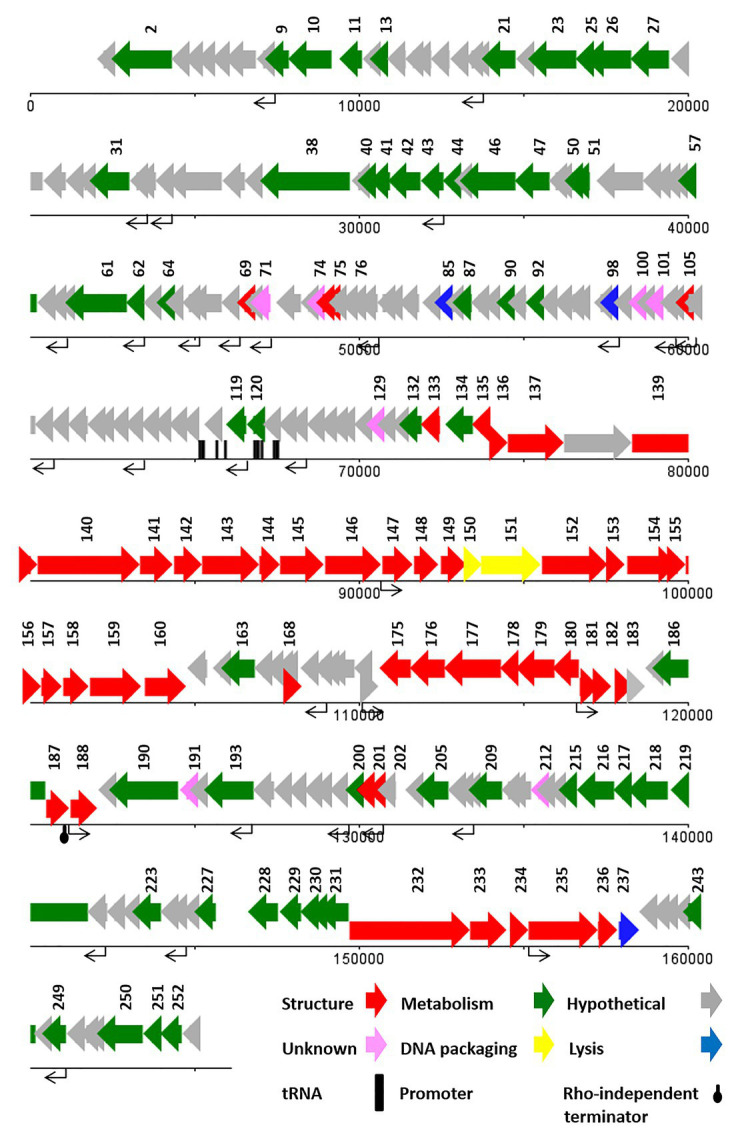
The linear genome map of phage vB_SdyM_006.

The genome of vB_SdyM_006 contains 252 ORFs (Supplementary Data 2) and nine tRNA coding regions (Supplementary Table S3). The only identified start codon was ATG. Ocher, Amber, and Opal stop codons were identified in 91, 50, and 111 ORFs, respectively. A BPROM search identified 31 promoters, with the consensus sequences of ATGTATAAT and TTTAAT at the −10 and −35 positions, respectively (the conserved bases were presented in bold; Supplementary Data 1). In addition, the only identified potential Rho-factor independent termination site was located after the gene encoding the inhibitor of the prohead protease (*gp187*; Figure 5).

With regard to the comprehensive genetic analysis of vB_SdyM_006 and the homology-based search of its 252 ORFs, the predicted ORFs could be clustered into five groups. Forty-five ORFs were predicted as structure proteins, nearly from *ORF133* to *ORF182* (Supplementary Data 2). Tail completion and sheath stabilizer protein, head completion protein, baseplate wedge subunit, baseplate wedge tail fiber connector, baseplate wedge subunit and tail pin, short tail fibers, fibritin, neck protein, tail sheath stabilization protein, tail sheath protein, tail tube protein, portal vertex of head, prohead core protein, prohead core scaffold protein, major capsid protein, capsid vertex protein, membrane protein, baseplate tail tube initiator, baseplate tail tube cap, baseplate hub subunit, and baseplate hub distal subunit all were detected in this region and have a high similarity rate to the respective predicted gene products of phages vB_PmiM_Pm5461, PM2, phiP4-3, and vB_MmoM_MP1 (Männistö et al., 1999; Oliveira et al., 2017; Morozova et al., 2018; Gkoutzourelas et al., 2020). Moreover, a small group of five genes was detected close to the end of the genome (from *ORF233* to *ORF237*) encoding different parts of the tail structure including long tail fiber proximal subunit, long tail fiber proximal connector, long tail fiber distal connector, long tail fiber distal subunit, and distal long tail fiber assembly catalyst which had 100% similarity to vB_PmiM_Pm5461 and phiP4-3 (Supplementary Data 2; Morozova et al., 2018; Gkoutzourelas et al., 2020).

Within the lysis functions, the *ORF98* encodes an endolysin with peptidase activity (conserved domain pfam13539). The *gp238* was identified as a Holin lysis mediator due to its high similarity to the respective predicted gene product of phage vB_PmiM_Pm5461. The *gp85* (lysis inhibition regulator) and *gp200* (rIII lysis inhibition accessory protein) are predicted to have the regulatory roles in the lysis pathway. It is worth mentioning that *ORF137* (baseplate hub + tail lysozyme) and *ORF157* (head core scaffold protein + protease) encodes bifunctional proteins whose contain either C‐ or N-terminal sequences and showed a relatively high similarity with those of the cell wall lysozymes.

Terminases, the proteins responsible for packaging the phage genome were detected almost in the middle of the genome (*ORF150* and *ORF151*), showing a ≥94% similarity to the small and large subunits of phage vB_MmoM_MP1 terminase (Figure 5 and Supplementary Data 2). Furthermore, the conserved domains of DNA_Packaging (pfam11053) and Terminase_6 (pfam03237) were identified in the small and large subunits of the terminase, respectively.

The predicted genes involved in the metabolic and regulatory functions were including several DNA-associated genes (DNA polymerase, helicase, primase, ligase, topoisomerase, and endonuclease proteins), RNA-associated genes (RNA polymerase, tRNA synthetase, ligase, endonuclease, and RNaseH proteins), different types of exonuclease, recombinase, anti-sigma factors, sigma factors, anaerobic NTP reductase, thioredoxin, kinase, host translation inhibitors, and several other genes. Most of the predicted proteins showed a high identity (≥90%) with the counterpart proteins of *Tevenvirinae* subfamily of phages while some others had no similarity (*gp71*, *gp74*, *gp100*, *gp129*, *gp191*, and *gp212*; Supplementary Data 2).

Based on the BLASTN analysis, the genome sequence of vB_SdyM_006 had 98% (97% query coverage) and 97% (73% query coverage) similarity to the genome sequences of *Proteus* phages, phiP4-3 and vB_PmiM_Pm5461, respectively. Moreover, the sequences alignment of these three phages using Gepard software showed a higher similarity between vB_SdyM_006 and phiP4-3 than vB_SdyM_006 and vB_PmiM_Pm5461 (Figure 4). Furthermore, the relatedness of vB_SdyM_006 and other phages with a high degree of homology was determined using Easyfig software (Supplementary Figure S3) and comparison of the basic genomic properties of the isolated phage of vB_SdyM_006 with other phages were summarized in Supplementary Table S4.

The CoreGene analysis showed that vB_SdyM_006 shared ~84% similarity with that of the encoded proteins of the mentioned phages above (score >70), including 111 hypothetical proteins and 101 known proteins with different functions. These protein-coding genes were spread out all along the genome and were not restricted to any particular region (Figure 4 and Supplementary Data 3).

#### Genome Analysis of vB_SsoS_008

**Figure 6:**
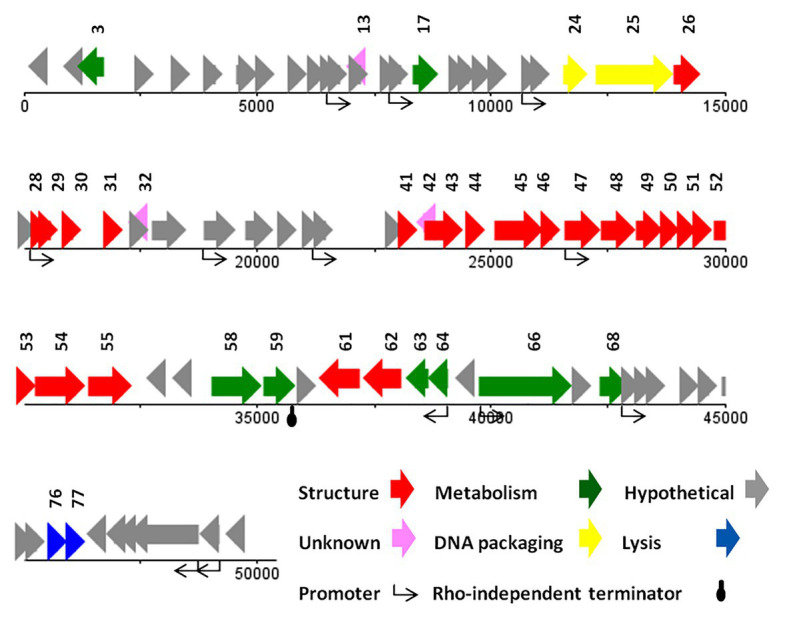
The linear genome map of phage vB_SsoS_008.

The genome of vB_SsoS_008 contained 83 putative ORFs, of which the function of 33 ORFs were predicted (Supplementary Data 2), and the other 50 ORFs were assigned as hypothetical proteins in which 47 ORFs had similarities with the hypothetical proteins of bacteriophages vB_EcoS_SH2, Sfin-1, T1, SH6, and phi2457T while the other 3 ORFs were evidently unique to UAB_Phi87 and showed no similarity with the already deposited sequences. Twelve sequences with conserved consensus sequences of gTtTAatAT (−10) and TTgCaA (−35) were identified as promoter and were distributed throughout the phage genome (the conserved bases were presented in capital letter; Supplementary Data 1). All of the ORFs started with an ATG codon, with Opal (36 ORFs), Ochre (30 ORFs), and Amber (17 ORFs) stop codons. Only one Rho-independent terminator was identified by FindTerm (Figure 6). The genome of vB_SsoS_008 contained no tRNA or pseudo-tRNA genes.

The vB_SsoS_008 ORFs were encoding known protein that can be classified into five functional groups. The structural group contained 21 proteins including portal protein (gp26), capsid proteins (gp28-31), tail proteins (gp41, 43–55, 61, and 62). All of the structural proteins showed a relatively high to high similarity (85–100%) with the respective predicted gene products of phages B_EcoS_SH2, Sfin-1, T1, SH6, and phi2457T, except *gp46* in which only 58% similarity was observed to the tail fibers protein of *Shigella* phage Sfin-1. Detection of pfam05939 conserved domain (Phage_min_tail) in this gene approved the function of *gp46* as the tail fibers. The second group includes eight proteins predicted to be associated with nucleotide metabolism and its regulation. The product of these genes facilitate genome replication, transcription, and translation. These proteins are DNA methylase (gp3), kinase (gp17), nuclease (gp58), recombination protein (gp59), DNA primase (gp63) and primase (gp64), helicase (gp66), and methyltransferase (gp68) which showed ≥80% similarity to the counterpart proteins of phages Sfin-1, T1, and phi2457T (Supplementary Data 2). The third group includes the necessary protein involved in the bacterial cell lysis process. The two genes, 76 and 77, are predicted to encode endolysin and spanin, respectively, and had 90% (query coverage of 65%) and 84% (query coverage of 79%) identity with their counterpart proteins of *Shigella* phage Sfin-1and *Shigella* phage SH6, respectively. Interestingly, holin gene was found neither close nor far from the lysine gene. The DNA packaging complex consisted of large (gp25) and small (gp24) subunits of terminase was categorized as the fourth group. The large subunit of this complex had a high identity (96%, query coverage of 100) while the small subunit had only 73% similarity (query coverage of 90%) with the counterpart proteins of the related phages (Supplementary Data 2).

BLASTN analysis of the phage vB_SsoS_008 genome showed ~91.2% (query coverage 90%), 91.7% (query coverage 84), and 90.5% (query coverage 96%) similarity with *Shigella* phage SH6, *Enterobacteria* phage T1, and *Shigella* phage Sfin-1, respectively. The CoreGenes analysis (score >70) revealed that vB_SsoS_008, *Shigella* phage SH6, *Enterobacteria* phage T1, and *Shigella* phage phi2457T had ~60% proteins in common including the structural, DNA packaging, metabolic, endolysin and hypothetical proteins (Figure 4 and Supplementary Data 3). In addition, the alignment of nucleotide sequences using Gepard software showed a high similarity between vB_SsoS_008 with *Shigella* phage SH6, *Enterobacteria* phage T1, and *Shigella* phage phi2457T (Figure 4). Furthermore, the relatedness of the vB_SsoS_008 and other phages with high homology was determined using the Easyfig (Supplementary Figure S4). The basic genomic properties of the isolated phage vB_SsoS_008 were compared to other phages and were summarized in Supplementary Table S4.

### Phylogenetic Analysis

**Figure 7:**
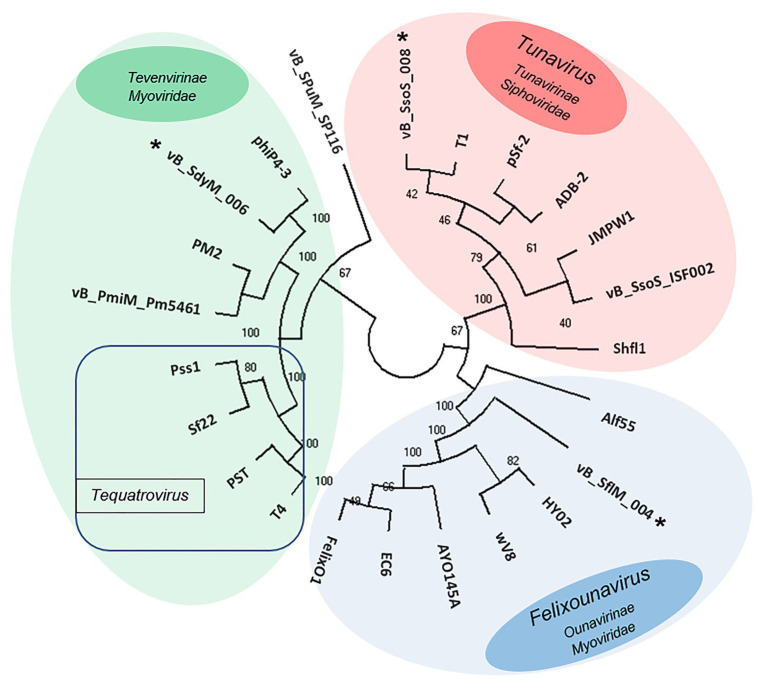
Unweighted pair group method with arithmetic mean (UPGMA) tree phylogenic analysis of the major capsids of phages vB _SflM_004, vB_SdyM_006, and vB_SsoS_008. The tree was rooted using the major capsid of *Salmonella enterica* serovar Pullorum lytic phage vB_SPuM_SP116 (Bao et al., 2019) as the out-group. The bootstrap percentages are shown next to each node. Phage groups as defined in the ICTV virus taxonomy (https://talk.ictvonline.org/taxonomy/) release are represented by different colors.

The phylogenetic relationship between the isolated phages and other similar phages available in online databases was studied using the construction of the phylogenic tree based on major capsid sequences that were identified in all of these phages (Figure 7). Both vB _SflM_004 and vB_SdyM_006 were clustered as a member of the *Myoviridae* family. However, their major capsid sequences were different enough to classify them into lower taxa levels in which vB _SflM_004 was clustered into *Felixounavirus* genus of *Ounavirinae* and vB_SdyM_006 was only classifiable to a subfamily level and as a member of the *Tevenvirinae*. The constructed phylogenic tree based on the major capsid sequences suggests that vB_SdyM_006 along with phiP4-3, PM2, and vB_PmiM_Pm5461 phages could be considered as a new genus in *Tevenvirinae* subfamily due to the considerable phylogenic distance with other related members such as those of *Tequatrovirus* genus. Moreover, the phylogenic analysis indicated that phage vB_SsoS_008 should be added to *Tunavirus* genus, *Tunavirinae* subfamily of *Siphoviridae* family.

## Discussion

### Independent Distribution of *Shigella* Phages From *Shigella* Bacterial Cells

There are many reports concerning isolation of *Shigella* phages from sources such as municipal wastewater, drainage ponds, and surface runoff after the regional occurrence of shigellosis, which could be due to the contact of *Shigella*-contaminated resource (such as the municipal wastewaters) with other water resources that usually are *Shigella* free (such as the runoff waters; Egoz et al., 1991; Barnell et al., 1996; Arias et al., 2006; He et al., 2012; Garner et al., 2017; Shahin et al., 2019c). In the present study, a number of *Shigella*-infecting phages were isolated from a large freshwater resource, the Yangtze River, and in an area with no prevalence of *Shigella*. This is especially interesting and important because no *Shigella* bacteria were recovered during the entire sampling period. Therefore, the relatively high abundance and diversity of *Shigella* phages could be an indication of their omnipresence in environments, especially in aquatic environments.

In general, the lipopolysaccharides of gram-negative bacteria include the O-antigen as the source of the bacterial diversity at the serotypes level, and the outer core as the source of diversity at the species level (Kropinski et al., 2007). In *Shigella* spp. the inner core is conserved (Brooke and Valvano, 1996). Since both vB _SflM_004 and vB_SsoS_008 phages infected all of the tested *S. sonnei* and *S. flexneri* isolates (with the exception of three isolates) in this study, it can be said that their function was independent of the O-antigen. The vB_SdyM_006 phage, however, only lysed *S.dysenteriae* isolates and had no effect on the isolates of other genera. Based on these observations, it can be inferred that the vB _SflM_004 and vB_SdyM_006 phages act as O-antigen-independent phages and are capable of lysing various species and serotypes, while vB_SdyM_006 is more specific and may only work on the serotypes of a certain species.

The presence of *Shigella* bacteria in various water resources has been recorded in different parts of the world. Connor et al. (2015) and Shahin et al. (2019c) acknowledged that neither *S. boydii* nor *S. dysenteriae* was the dominant endemic species in any country. However, *S. sonnei* is the dominant species in industrialized countries (WHO, 2005). *Shigella flexneri*, which has had a longer and more frequent history of presence, is also distributed worldwide (Shahin et al., 2019c). We expected in our observations to see coordination between the distribution and presence of the bacteria and of their infecting phages. Therefore, at first glance, it was expected that the frequency of *S. sonnei* and *S. flexneri* would be high in the studied aqueous environment (at least at the time of phage isolation). Nevertheless, despite using an enrichment step no *Shigella bacteria* were recovered during this research. Considering the proven specific nature of the phage-host interaction in many cases, a plausible hypothesis is that these phages have high stability in such environmental conditions and therefore remained dormant in the environment until the proper host reappears. In other words, *Shigella* and their infecting phages entered the environment and after a while, with the destruction of the bacterial host, the phages remained in the environment. However, given the very high abundance of the phages (estimated at 10^31^) on our planet and the bacteria in natural environment (Hendrix et al., 2002), as well as the culturability of only a very small percentage of bacterial species under laboratory conditions (Buchanan and Gibbons, 1974), another improbable but possible hypothesis is that there are other host(s) for vB _SflM_004, vB_SdyM_006, and vB_SsoS_008 phages. In other words, these phages may have infected some of the yet-to-be-cultivated bacteria.

Doore et al. (2019) hypothesized that *S. flexneri* might be the dominant species in the studied environment (water resources of Michigan, United States) in view of a high abundance of *S. flexneri* phages in those ecosystems. In our study, isolation of vB _SflM_004 without pre-enrichment (method II) indicates its higher frequency than that of the other two phages. The higher structural, host, and biological diversity in the isolated phages of the present study than the *Shigella* phages isolated from the Michigan and Nebraska aquatic environments could be justified due to the size of the studied water resource (the Yangtze River) and the repeated sampling at different times in the present investigation.

### Suitable Biological Properties for Stability in the Environmental

The thermos‐ and pH-stability tests demonstrated that for all of three phases (vB _SflM_004, vB_SdyM_006, and vB_SsoS_008) more than 50% of the infectivity was preserved at temperatures of −20 to 60°C and pHs of 6−10. Similar to our observation, it was reported frequently that *Shigella* phages have higher stability under alkaline conditions compared to acidic conditions (Jun et al., 2013; Hamdi et al., 2017; Shahin and Bouzari, 2018; Shahin et al., 2018; Doore et al., 2019). The absence of any significant changes in the phage titers at −20 to 40°C and in neutral to somewhat alkaline pH is an indication of the high stability of the phages in normal environments (neutral or slightly acidic/alkaline pH and ambient temperatures of ≤40°C).

One-step growth curve analysis and phage adsorption rate provide a comprehensive perspective of phage interactions with their bacterial host. In general, short adsorption time indicates a more powerful initial attachment behavior of a phage to its host surface, while shorter latent period and larger burst size are indications of a higher of lytic potency of a phage (Shahin et al., 2018). Therefore, in the case of the isolated *Shigella* phages in this study, it can be assumed that almost immediately after the introduction of an appropriate host cell to the environment, these phages identify the host cells, get attached, then replicate very fast and keep their titer high in the environment.

### The Isolated *Shigella* Phages Have Unique DNA Fingerprints

The use of restriction enzymes to determine the genome size of bacteriophages and to differentiate them from each other has a long history (Braun et al., 1989). The obtained DNA fingerprint profiles indicated that vB _SflM_004 and vB_SdyM_006 phages were different from each other and belong to two different groups despite both of them being a member of *Myoviridae* phages as it proved through the whole genome sequencing. Moreover, a comparison of these profiles with the already reported *Shigella* phages such as pSf-1, pSf-2, Shfl1, vB_SflS-ISF001, vB_SsoS-ISF002, and vB-SdyS-ISF003 shows a clear difference. Therefore, it can be concluded that the DNA fingerprint profiles can be used as a genome-based typing method to identify similar phages. Our results demonstrate that rapidness, cost-effectiveness, proper degree of sensitivity, in addition to not using any sophisticated laboratory equipment are the advantages of this test and makes it an appropriate method for preliminary analysis of phage diversity.

### High Diversity of *Shigella* Phages

Although vB _SflM_004 and vB_SdyM_006 phages were both of the *Myoviridae* family, there were substantial phylogenetic differences to placed vB _SflM_004 in the *Felixounavirus* genus, *Ounavirinae* subfamily of *Myoviridae* family. Moreover, although vB_SdyM_006 together with Phi4-3, PM2, Pm5461, CGG4-1, PEi20, and PST, and T4 phages were phylogenetically placed in the *Tevenvirinae* subfamily, these phages were sufficiently different from Pss1, Sf 22, PST14 phages (of the *Tequatrovirus* genus) to be clustered in a new genus together with phages closely related to it such as Phi4-3, PM2, and PM5461 (Männistö et al., 1999; Oliveira et al., 2017; Morozova et al., 2018; Gkoutzourelas et al., 2020). Several phage families are capable of infecting *Shigella* bacteria. According to the latest online version of the ICTV (2018), totally there are 24 *Shigella*-infecting phages in the *Siphoviridae* (5 phages), *Podoviridae* (5 phages), *Myoviridae* (13 phages), and *Ackermannviridae* (1 phage) families. Through searching the public databases, a total 35 phages were found as the *Shigella*-infecting phages between 2016 and 2018. However, since then until January 2020, another 73 complete sequences of *Shigella* phages (39 *Myoviridae*, 24 *Siphoviridae*, 8 *Podoviridae*, and 2 *Ackermannviridae*) were registered at NCBI.^10^ In the present study, isolation of more phages from *Myoviridae* family and even without using pre-enrichment in the case of vB_SflM_004, is in agreement with the general pattern mentioned above, that is a higher frequency of bacteriophages from *Myoviridae* family in the previous studies.

In conclusion, isolation of the three types of lytic phages while no *Shigella* bacteria were recovered implies that the presence of phages in an environment could be completely independent of the presence of their target host bacteria. Such observation is highly likely due to a high level of stability of these phages in the environment, or in a less-likely hypothesis, due to the phages having other host(s) among the yet-to-be-cultivated bacteria. Furthermore, *Shigella* phage isolation using methods with or without enrichment also demonstrated coordination in the distribution of these phages in freshwater and other environments. The genomic and bioinformatics analyses did not identify any genes involved in the lysogenic cycles in the genome sequences of the three isolated phages, and no plaques suspected of harboring lysogenic phages were detected during the tests. Both of these observations indicate the absolute linearity of these phages and, given the desirability of their other biological properties, they can be introduced as suitable antibacterial candidates for bio-control of *Shigella* in different conditions.

## Data Availability Statement

The datasets presented in this study can be found in online repositories. The names of the repository/repositories and accession number(s) can be found in the article/Supplementary Material.

## Author Contributions

KS and RWa designed the research. KS, MB, and AH contributed to data analysis and drafted the manuscript. MB, LZ, HB, MM, MP, and TH performed the laboratory phage works, DNA extraction and sequencing. All authors contributed to the article and approved the submitted version.

### Conflict of Interest

The authors declare that the research was conducted in the absence of any commercial or financial relationships that could be construed as a potential conflict of interest.

## Footnotes

^1^http://opal.biology.gatech.edu/genemark/gmhmmp.cgi

^2^https://www.ncbi.nlm.nih.gov/orffinder/

^3^http://web.expasy.org/translate/

^4^http://web.expasy.org/compute_pi/

^5^https://blast.ncbi.nlm.nih.gov/Blast.cgi

^6^https://toolkit.tuebingen.mpg.de/#/tools/hhpred

^7^http://pfam.xfam.org/search#tabview=tab1

^8^http://www.ebi.ac.uk/interpro/search/sequence-search

^9^https://ncbi.nlm.nih.gov

^10^https://www.ncbi.nlm.nih.gov/nuccore
