# Supplementary material for: Biodiversity of New Lytic Bacteriophages Infecting Shigella spp. in Freshwater Environment
Source: Front Microbiol. 2021 Feb 17;12:619323. doi: 10.3389/fmicb.2021.619323 (PMC7925395; doi:10.3389/fmicb.2021.619323)
Supplement: Supplementary file 1 [file Data_Sheet_1.pdf]

Biodiversity of new lytic bacteriophages infecting *Shigella* spp. in fresh water environment

Khashayar Shahin[1], Mohadeseh Barazandeh[1], Lili Zhang[1], Abolghasem Hedayatkhah[2], TaoHe[1], Hongduo Bao[1], Mojtaba Mansoorianfar[3], Maoda Pang[1], Heye Wang[1], Ruicheng Wei[1], Ran Wang[1]

[1] Institute of Food Safety and Nutrition, Key Lab of Food Quality and Safety of Jiangsu Province-State Key Laboratory Breeding Base, Jiangsu Academy of Agricultural Sciences, Nanjing, 210014, China

[2] School of Pharmacy and Biomedical Sciences, CHIRI Biosciences, Curtin University, GPO Box U1987, Perth, WA 6845, Australia

[3] Nanjing Forestry University

Table S1.

The bacterial strains used in this study, and the host range and efficiency of plating (EOP) for the three isolated bacteriophages

Bacterial species	Source	vB_SflM_004		vB_SdyM_006		vB_SsoS_008	
		Spot test	EOP	Spot test	EOP	Spot test	EOP
Shigella dysenteriae	PTCC 1188	–		++	0.93 ± 0.06	–	
***Shigella dysenteriae* (S.d.f1)**	Vegetables	–		++	1.00	–	
***Shigella dysenteriae* (S.d.f2)**	Vegetables	–		++	0.77 ± 0.05	–	
Shigella sonnei	ATCC 9290	+	0.14 ± 0.08	–		++	0.88 ± 0.06
***Shigella sonnei* (w8)**	Ready to eat food	–		–		++	1.00
***Shigella sonnei* (w9)**	Vegetables	+	0.25 ± 0.03	–		++	0.68 ± 0.05
***Shigella sonnei* (w44)**	Ready to eat food	–		–		+	0.35 ± 0.04
***Shigella sonnei* (Sh.s-3)**	Vegetables	+	0.29 ± 0.05	–		+	0.23 ± 0.05
***Shigella sonnei* (Sh.s-26)**	Meat	+	0.33 ± 0.06	–		++	0.70 ± 0.05
***Shigella sonnei* (Sh.s-77)**	Vegetables	–		–		+	0.40 ± 0.01
Shigella flexneri	ATCC 12022	++	0.97 ± 0.01	–		++	0.65 ± 0.05
Shigella flexneri	PTCC 1234	++	0.90 ± 0.05	–		+	0.12 ± 0.07
Shigella flexneri	PTCC 1865	++	0.92 ± 0.03	–		+	0.22 ± 0.03
***Shigella flexneri* (w7)**	Ready to eat food	++	1.00	–		++	0.56 ± 0.02
***Shigella flexneri* (w16)**	Vegetables	++	0.70 ± 0.09	–		++	0.43 ± 0.08
***Shigella flexneri* (w19)**	Vegetables	++	0.75 ± 0.05	–		+	0.30 ± 0.04
***Shigella flexneri* (w27)**	Vegetables	++	0.47 ± 0.06	–		++	0.54 ± 0.01
***Shigella flexneri* (W2)**	Vegetables	++	0.39 ± 0.02	–		++	0.50 ± 0.08
***Shigella flexneri* (Sh.f-8)**	Vegetables	++	0.41 ± 0.02	–		+	0.17 ± 0.02
Escherichia coli	ATCC 25922	–		–		–	
Escherichia coli	Urinary Tract Infection	–		–		–	
Escherichia coli	Bedsore	–		–		–	
Klebsiella pneumoniae	ATCC 13883	–		–		–	
Streptococcus pneumoniae	ATCC 49619	–		–		–	

Pseudomonas aeruginosa	ATCC 27853	–	–	–
Pseudomonas aeruginosa	Burn Wound Infection	–	–	–
Enterobacter cloacae	Bedsore	–	–	–
Enterococcus faecalis	ATCC 29212	–	–	–
Salmonella typhimurium	ATCC 14028	–	–	–
Proteus mirabilis	ATCC 43071	–	–	–
Staphylococcus saprophyticus	ATCC 15305	–	–	–
Staphylococcus epidermidis	ATCC 12228	–	–	–
Staphylococcus aureus	ATCC 35933	–	–	–
Staphylococcus aureus MRSA	ATCC 33591	–	–	–
Bacillus cereus	ATCC 11778	–	–	–
Bacillus subtilis	ATCC 12711	–	–	–
Enterobacter aerogene	ATCC 13048	–	–	–
Streptococcus pyogenes	ATCC 19615	–	–	–
Proteus mirabilis	ATCC 43071	–	–	–

+ +, clear plaque; +, cloudy plaque; −, no plaque. The EOP (efficiency of plating) values were shown as the mean of three repeats ± standard deviation (SD)

Table S2.

Categorization of the efficiency of plating (EOP) of the *Shigella* phages against different isolates of *Shigella* spp..

	**vB_SflM_004**	**vB_SdyM_006**	**vB_SsoS_008**
***S. dysenteries* (n=3)**			
EOP ≥ 0.5	–	3	–
0.1 ≤ EOP < 0.5	–	0	–
0.001 < EOP < 0.1	–	0	–
***S. flexneri* (n=9)**			
EOP ≥ 0.5	6	–	4
0.1 ≤ EOP < 0.5	3	–	5
0.001 < EOP < 0.1	0	–	0
***S. sonnei* (n=7)**			
EOP ≥ 0.5	0	–	4
0.1 ≤ EOP < 0.5	4	–	3
0.001 < EOP < 0.1	0	–	0

Table S3.

The predicted tRNA coding sequences of vB_SdyM_006 genome

tRNA	Strand	Position	Anticodon	Amino acid
**1**	Complement	65128..65203	TTC	Glu
**2**	Complement	65211..65302	TGA	Ser
**3**	Complement	65651..65726	GTC	Asp
**4**	Complement	65920..65994	TCC	Gly
**5**	Complement	66799..66874	CAT	Ile2
**6**	Complement	66882..66966	GTA	Tyr
**7**	Complement	67012..67088	TCT	Arg
**8**	Complement	67403..67477	CCA	Trp
**9**	complement	67479..67562	GTT	Asn

Table S4.

Comparison of the basic genomic properties of phages vB_SflM_004, vB_SdyM_006 and vB_SsoS_008 with other similar phages

	% identity	Genome length	GC-content	Total/identified ORF	No. of tRNA	Accession no.
**vB_SflM_004**	100	85,887	38.6	135/48	0	MK295205
**WV8**	94.2	88487	38.7	137/20	18	EU877232
**phage 11**	94.2	88771	38.5	148/47	26	KP869109
**phage 12**	94.2	88632	38.4	132/21	26	KP869110
**BPS15Q2**	94.1	89817	38.6	130/45		KX405003
**vB_SdyM_006**	100	166,138	31.5	252/105	9	MK295204
**phiP4-3**	97	167849	31.4	270/119	8	MG696114
**vB_PmiM_Pm5461**	86	161989	31.5	252/119	8	KP890823
**vB_SsoS_008**	100	50,414	46.5	83/34	0	MK335533
**T1**	91.73	48836	46.5	78/23	0	AY216660
**phi2457T**	91	50219	46.6	80/21	0	MH917278
**SH6**	91	50552	46.6	82/26	0	NC_047785

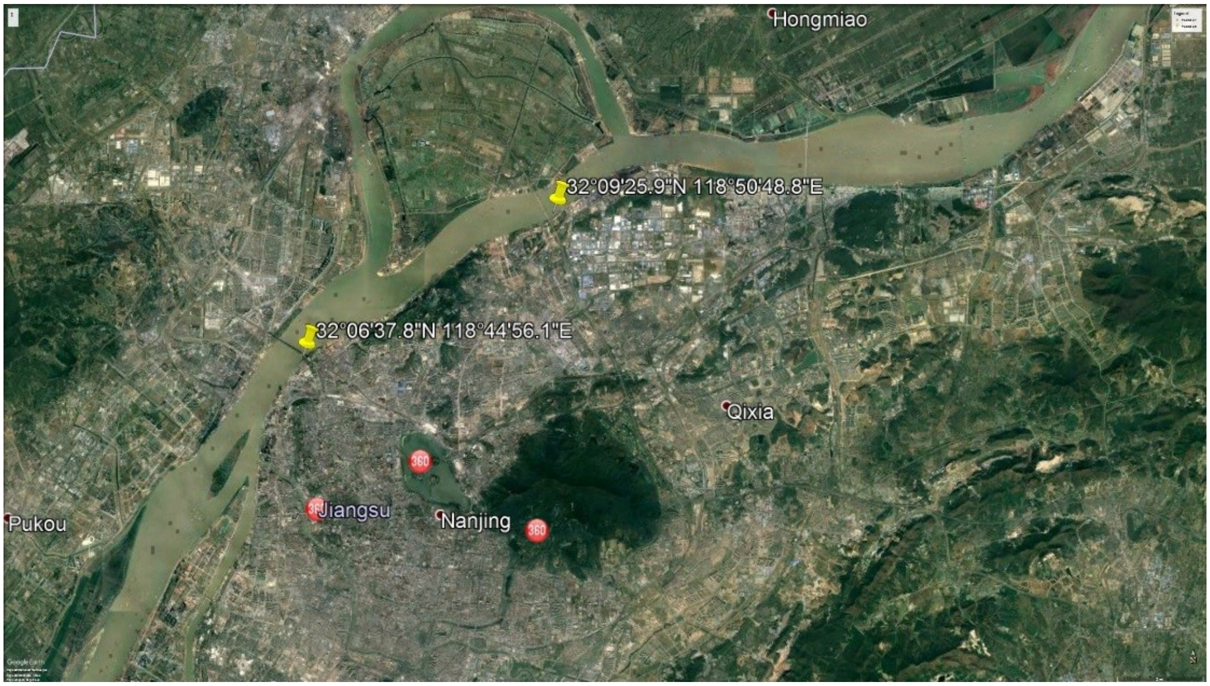

Fig S1. Sampling sites along the Yangtze River. The Yellow pins represent latitude: 32°06'37.8"N and longitude: 118°44'56.1"E and latitude: 32°09'25.9"N and longitude: 118°50'48.8"E

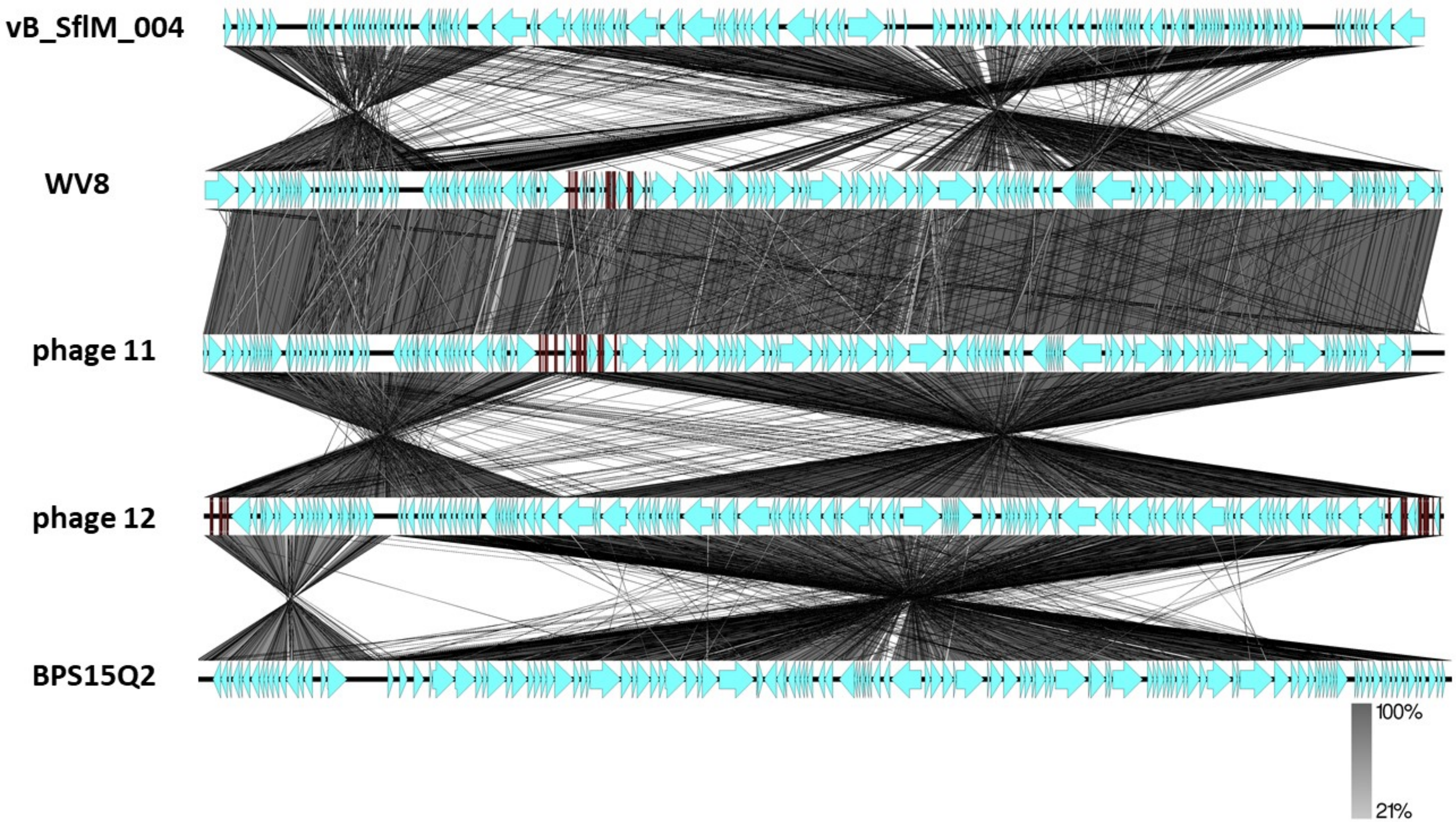

Fig S2. BLASTp comparison of *Shigella* vB_SflM_004 and the most closet phages. The figure was constructed using EasyFig.

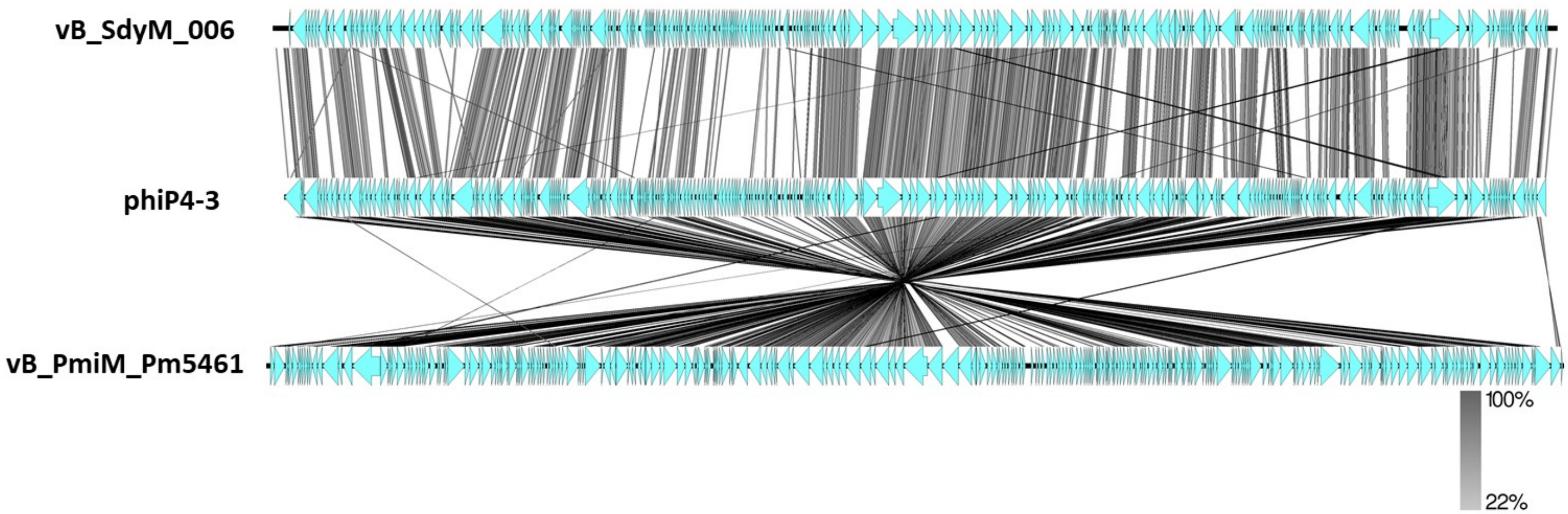

Fig S3. BLASTp comparison of *Shigella* vB_SdyM_006 and the most closet phages. The figure was constructed using EasyFig

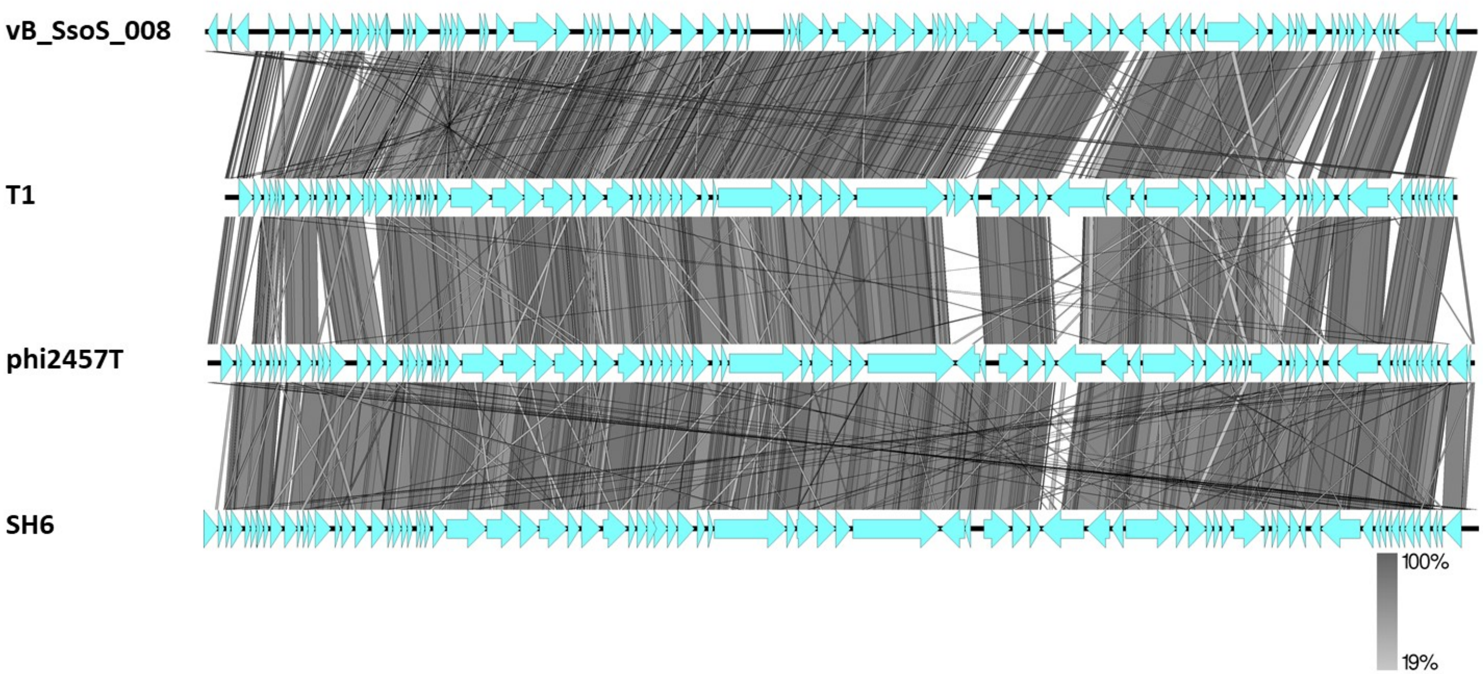

Fig S4. BLASTp comparison of Shigella vB_SsoS_008 and the most closet phages. The figure was constructed using EasyFig.
