# Supplementary material for: Biodiversity of New Lytic Bacteriophages Infecting Shigella spp. in Freshwater Environment
Source: Front Microbiol. 2021 Feb 17;12:619323. doi: 10.3389/fmicb.2021.619323 (PMC7925395; doi:10.3389/fmicb.2021.619323)
Supplement: Supplementary file 2 [file Data_Sheet_2.pdf]

Promotor of phage vB_SflM_004

	position	-10	-35
**1**	44640	TTTTATAAT	TTGACA
**2**	58710	AGGTATTAT	TTGAGA
**3**	52297	TTTTATACT	TTGACA
**4**	61088	TCATATAAT	TTGATT
**5**	36774	TTTTATTCT	TTTACT
**6**	7194	TTTTAGAAT	TTGATA
**7**	70277	TTTTATTAT	TTTATA
**8**	73900	TTGTATGAT	TTGACA
**9**	69409	TTTTAAAAT	TTGAAA
**10**	81365	GGTTATAAT	TTGACC
**11**	73309	AGTTATAGT	TTAAAA
**12**	16863	CTGTATCTT	TTAACT
**13**	80644	TTTTAGATT	TTGATT
**14**	50750	AACTAAAGT	TTTACA
**15**	76849	GATTAAAGT	TTGATT
**16**	63223	TGCTAAGAT	TTCACA

Promotor of phage vB_SdyM_006

	position	-10		-35
**1**	7199	TTTTATAAT		TTGTAA
**2**	13495	TGCTATAAT		TTCTAG
**3**	23291	TGTTATTAT		TTCAAA
**4**	24211	AATTATAAT		TTTTAA
**5**	32593	CATTATAAT		TTAAAG
**6**	40886	TTCTATAAT		TTAAAA
**7**	43510	TAGTATAAT		TTGTAG
**8**	44855	CCTTATAAT		TTTAAG
**9**	46330	CCTTATAAT		TTGTAA
**10**	47337	TGATATAAT		TTGTAA
**11**	49343	GTTTATGAT		TTGCAT
**12**	57794	GGTGATAAT		TTAAAT
**13**	59163	TTTTATCAT		TTCCAG
**14**	59564	CACTATTAT		TTGTAA
**15**	60649	ATTCATAAT		TTGAAT
**16**	62984	TGGTATAAT		TTTCAA
**17**	66585	ATG**TAT**AA**T**		**TT**TA**A**T
**18**	68320	AATTATAAT		TTGTAT
**19**	90708	TTATATAAT		TTAAAA
**20**	108854	CGGTATAAT		TTCAAG
**21**	110236	TGATATTAT		TTTAAT
**22**	116646	TAGTATAAT		TTGTAA
**23**	120480	TGTTATAAT		TTTAAT
**24**	126715	TTTTATAAT		TTTAAT
**25**	129414	ATCTATAAT		TTTAAA
**26**	130580	CTGTATGAT		TTAAAT
**27**	133173	CATTATAAT		TTGCAA
**28**	142336	TATTATTAT		TTTCAG
**29**	144627	TTATATAAT		TTGGAT
**30**	154613	ATATATAAT		TTTAAT
**31**	161520	TGATATGAT		TTCTAT

Promotor of phage vB_SsoS_008

	position	-10	-35
**1**	6467	ATGTAACAT	TTGCGA
**2**	7769	GTCTATGAT	TTGCTA
**3**	10884	TTGTATAAT	TTCCAA
**4**	15294	CTCTAGGAT	TTGCAA
**5**	17696	GTTTAATAT	TTGCAA
**6**	21050	CTATATGAT	TTCCCA
**7**	26544	CTGTAGAAT	TTGCGA
**8**	38117	TTCTAAGAT	TTCCTA
**9**	39685	GTGTATTAT	TTGCTA
**10**	42929	GTTTAAAAT	TTCCCA
**11**	48741	GTTTAATAT	TTGCTA
**12**	49049	TTTTAACAT	TTTCAA
