# Supplementary material for: Biodiversity of New Lytic Bacteriophages Infecting Shigella spp. in Freshwater Environment
Source: Front Microbiol. 2021 Feb 17;12:619323. doi: 10.3389/fmicb.2021.619323 (PMC7925395; doi:10.3389/fmicb.2021.619323)
Supplement: Supplementary file 3 [file Data_Sheet_3.pdf]

The predicted ORFs of phage **vB_SflM_004**, their putative function and consereved domain

	CDS_POSITION	Strand	Best BLAST hit (organism)	Coverage (%)	E-value	Identity (%)		Consereved domain	
1	120..623	+	hypothetical protein [Salmonella phage FSL SP-107]	100%	2.00E-120	98%	AGF89482.1		
2	1008..1430	+	hypothetical protein [Escherichia phage wV8]	100%	2.00E-95	96%	YP_002922811.1		
3	1420..1884	+	hypothetical protein [Salmonella phage FSL SP-107]	100%	1.00E-104	95%	AGF89485.1		
4	1898..2446	+	hypothetical protein [Escherichia phage vB_EcoM-VpaE1]	100%	7.00E-125	93%	YP_009147295.1		
5	2846..3310	+	hypothetical protein [Escherichia phage JH2]	100%	1.00E-105	96%	YP_009219568.1		
6	3324..3872	+	hypothetical protein [Escherichia phage vB_EcoM_Alf5]	100%	7.00E-131	97%	YP_009288208.1		
7	5997..6245	-	hypothetical protein [Salmonella phage FSL SP-107]	100%	2.00E-54	98%	AGF89487.1		
8	6314..6577	-	hypothetical protein [Escherichia phage wV8]	100%	1.00E-53	95%	YP_002922807.1		
9	6639..6959	-	hypothetical protein [Escherichia phage HY02]	86%	5.00E-40	73%	YP_009204928.1		
10	6922..7179	-	hypothetical protein [Enterobacteria phage KhF1]	97%	6.00E-44	86%	ANZ51930.1		
11	7530..7946	-	hypothetical protein [PHAGE_Escher_HY02]						
12	8467..8700	-	hypothetical protein [PHAGE_Escher_vB_EcoM_Alf5]						
13	8764..9327	-	hypothetical protein [PHAGE_Escher_JH2]						
14	9413..9661	-	hypothetical protein [PHAGE_Escher_phiSUSP2]						
15	9752..9949	-	prohead assembly scaffold protein [PHAGE_Salmon_BPS15Q2]						
16	10050..10457	-	hypothetical protein [Salmonella phage BPS17W1]	100%	1.00E-88	96%	AUM59093.1		
17	10546..10818	-	hypothetical protein [Salmonella phage FSL SP-012]	100%	1.00E-61	100%	AGF88919.1		
18	10910..11248	-	tail tube protein [PHAGE_Salmon_BPS15Q2]						
19	11242..11538	-	tail sheath monomer [PHAGE_Salmon_BPS15Q2]						
20	11627..11932	-	hypothetical protein [Shigella phage Sf13]	100%	6.00E-66	100%	ATE85853.1		
21	12204..12461	-	hypothetical protein [Salmonella phage FSL SP-107]	96%	1.00E-44	89%	AGF89438.1		
22	12553..12933	-	hypothetical protein [Escherichia phage JH2]	100%	7.00E-84	95%	YP_009219453.1		
23	13184..13402	-	hypothetical protein [Salmonella phage FSL SP-107]	94%	2.00E-39	94%	AGF89496.1		
24	13923..14717	-	putative phosphatase [Escherichia phage EC6]	100%	0	97%	YP_009151369.1	Macro_Poa	cd02901
25	14718..14918	-	hypothetical protein [Escherichia phage JH2]	100%	4.00E-41	100%	YP_009219456.1		
26	15142..15468	-	tail assembly protein [PHAGE_Salmon_BPS15Q2]						
27	15478..15747	-	hypothetical protein [PHAGE_Escher_vB_EcoM_Alf5]						
28	15744..15941	-	o-spanin [Salmonella phage BPS15Q2]	100%	2.00E-38	97%	YP_009324690.2		
29	15907..16254	-	i-spanin [Salmonella phage Mushroom]	100%	2.00E-77	97%	AJF40654.1		
30	16307..16771	-	hypothetical protein [Escherichia phage HY02]	100%	1.00E-105	99%	YP_009204908.1		
31	16783..17478	-	polynucleotide kinase [Salmonella phage Mushroom]	100%	1.00E-167	97%	AJF40656.1		
32	18105..19244	-	rIIB protein [Salmonella phage FSL SP-010]	100%	0	96%	AGF88670.1	valS	PRK14900
33	19324..21696	-	rIIA lysis inhibitor [Enterobacteria phage KhF1]	100%	0	98%	ANZ51857.1	HtpG	COG0326
34	21920..22255	-	hypothetical protein [Escherichia phage HY02]	100%	6.00E-76	100%	YP_009205027.1		

35	22252..22467	-	DNA polymerase [Salmonella phage SHP1]	64%	2.00E-03	42%	ASJ79448.1		
36	22521..24335	-	nictotinate phosphoribosyltransferase [Escherichia phage vB_Ec	100%	0	97%	YP_009200868.1	nadV	PHA02594
37	24354..24845	-	HNH endonuclease [Salmonella phage Meda]	100%	1.00E-115	98%	AXY86265.1		
38	24854..25735	-	putative ribose-phosphate pyrophosphokinase [Escherichia phag	100%	0	97%	YP_009288313.1	PrsA	COG0462
39	25748..26026	-	hypothetical protein [Escherichia phage EC6]	100%	8.00E-63	100%	YP_009151351.1		
40	26019..26534	-	hypothetical protein [Escherichia phage vB_EcoM_AYO145A]	100%	1.00E-122	97%	YP_009200865.1		
41	26586..26906	-	hypothetical protein [Escherichia phage EC6]	100%	3.00E-68	98%	YP_009151349.1		
42	26909..27166	-	hypothetical protein [Escherichia phage wV8]	100%	2.00E-53	100%	YP_002922914.1		
43	27133..27921	-	anaerobic nucleoside-triphosphate reductase subunit [Salmonell	57%	6.00E-107	99%	AJF40541.1	NrdG	TIGR02491
44	27982..28377	-	tail tube protein [PHAGE_Escher_vB_EcoM_Alf5]	100%	2.00E-91	98%	YP_009288305.1		
45	28421..28693	-	hypothetical protein [Shigella phage Sf17]	87%	3.00E-23	66%	AUV62969.1		
46	28669..28824	-	unknown [Salmonella phage FelixO1]	100%	3.00E-24	90%	AAQ14733.1		
47	28848..30992	-	anaerobic ribonucleoside-triphosphate reductase [Escherichia co	100%	0	99%	WP_113809211.1	NrdD	TIGR02487
48	31041..31247	-	unknown	100%	6.00E-39	100%	WP_113809212.1		
49	31482..32555	-	ribonucleoside triphosphate reductase beta chain [Escherichia p	100%	0	99%	YP_009147382.1	RNRR2	cd01049
50	32552..32809	-	hypothetical protein [Escherichia phage JH2]	100%	2.00E-53	95%	YP_009219484.1		
51	32865..35099	-	Ribonucleoside triphosphate reductase, alpha chain [Salmonella	100%	0	99%	NP_944991.1	ATP-cone	pfam03477
52	35146..35484	-	hypothetical protein [Salmonella phage vB_SPuM_SP116]	100%	9.00E-76	98%	YP_009146352.1		
53	35477..35800	-	hypothetical protein [Salmonella phage FelixO1]	100%	9.00E-75	100%	NP_944987.2		
54	35781..36536	-	phosphoribosyl-ATP pyrophosphohydrolase [Salmonella phage	100%	0	98%	AUM59523.1	PRA-PH	pfam01503
55	36529..36759	-	hypothetical protein [Enterobacteriaphage UAB_Phi87]	100%	1.00E-45	100%	YP_009150138.1		
56	36781..37278	-	NAD synthetase [Salmonella phage BPS15Q2]	100%	4.00E-118	99%	YP_009324660.1	GIY-YIG_S	cd10444
57	37268..37774	-	HNH endonuclease [Citrobacter phage Mijalis]	95%	4.00E-117	99%	ARB06520.1	HNH_3	pfam13392
58	37752..38798	-	putative exodeoxyribonuclease [Escherichia phage wV8]	100%	0	97%	YP_002922895.1	PHA00439	PHA00439
59	38861..39718	-	hypothetical protein [Escherichia phage wV8]	100%	0	99%	YP_002922894.1		
60	40193..42178	-	putative phage DNA primase/helicase [Salmonella phage vB_SI	100%	0	99%	YP_009146344.1	GP4d_helic	cd01122
61	42171..42365	-	hypothetical protein [Escherichia phage EC6]	100%	1.00E-35	98%	YP_009151324.1		
62	42380..43123	-	putative deoxynucleotide monophosphate kinase(dNMP) [Enter	100%	4.00E-177	97%	AWD91383.1	1	PHA02575
63	43185..43997	-	minor tail protein [Salmonella phage BPS17L1]	100%	0	93%	AUM59533.1		
64	43999..44421	-	unknown	100%	1.00E-98	100%	WP_015973568.1		
65	44679..47378	+	putative DNA polymerase [Escherichia phage vB_EcoM_Alf5]	100%	0	99%	YP_009288280.1	DNA_pol_A	cd08643
66	47498..47638	+	hypothetical protein [Citrobacter phage Moogle]	100%	2.00E-43	100%	YP_009145681.1		
67	47841..48092	+	hypothetical protein [Shigella phage Sf15]	60%	3.00E-28	96%	ATE85990.1		
68	48692..48898	+	hypothetical protein [Citrobacter phage Moogle]	100%	3.00E-36	90%	YP_009145684.1		
69	50759..51340	+	hypothetical protein [Citrobacter phage Moogle]	100%	1.00E-131	93%	YP_009145686.1		
70	51343..51783	+	hypothetical protein [Shigella phage Sf17]	100%	5.00E-103	99%	AUV62937.1		
71	52330..52713	+	hypothetical protein [Citrobacter phage Moogle]	100%	9.00E-78	90%	YP_009145688.1	PRK15239	PRK15239
72	52914..53213	+	transcriptional regulatory protein [PHAGE_Escher_phiSUSP1]					HTH_37	pfam13744
73	53340..53570	+	nucleoside triphosphate hydrolase [PHAGE_Escher_vB_EcoM	100%	3.00E-45	92%	ALA06866.1		

74	53555..53947	+	hypothetical protein [Shigella phage Sf15]	93%	2.00E-76	92%	ATE86001.1		
75	54100..54381	+	hypothetical protein [Shigella phage Sf18]	84%	2.00E-45	91%	ATE86284.1		
76	54345..54548	+	hypothetical protein [Shigella phage Sf16]	100%	3.00E-35	93%	ATE86206.1		
77	54930..55253	+	dihydrofolate reductase [Shigella phage Sf18]	99%	2.00E-59	88%	ATE86282.1	DHFR	cd00209
78	55250..56140	+	thymidylate synthase [Shigella phage Sf16]	84%	5.00E-173	93%	ATE86203.1	Thymidylat_	pfam00303
79	56335..56622	-	putative holin [PHAGE_Escher_phiSUSP1]						
80	56622..56801	-	hypothetical protein [Citrobacter phage Moogle]	100%	8.00E-27	84%	YP_009177305.1		
81	56888..57766	-	tail fiber protein [Shigella phage Sf19]	99%	6.00E-115	64%	AUV56273.1		
82	57900..58241	-	tail fiber protein [PHAGE_Escher_wV8]					gp37_C	pfam12604
83	58405..58791	-	tail fiber protein [PHAGE_Escher_vB_EcoM_AYO145A]						
84	59135..59440	-	tail fiber protein [PHAGE_Escher_phiSUSP1]						
85	59486..60481	-	tail fiber protein [PHAGE_Escher_wV8]						
86	60678..61076	-	hypothetical protein [Shigella phage Sf15]	64%	1.00E-55	100%	ATE86010.1		
87	61489..62061	-	baseplate assembly protein [Shigella phage Sf18]	83%	1.00E-87	86%	ATE86410.1	Baseplate_J	pfam04865
88	62453..62641	+	hypothetical protein [Escherichia phage vB_EcoM_Alf5]	72%	7.00E-21	94%	YP_009288277.1		
89	62740..63144		hypothetical protein [Escherichia phage wV8]	79%	5.00E-70	98%	YP_002922853.1	DUF3277	pfam11681
90	63160..63468	-	hypothetical protein [Shigella phage Sf17]	100%	3.00E-60	96%	AUV62911.1	DUF3383	pfam11863
91	63472..63816	-	tail sheath [Salmonella phage Meda]	97%	7.00E-56	83%	AXY86327.1		
92	63818..64054	-	tail sheath [Salmonella phage Meda]	75%	1.00E-15	69%	AXY86327.1		
93	64269..64556	-	hypothetical protein [Citrobacter phage Moogle]	77%	8.00E-46	96%	YP_009145715.1		
94	64553..64828	-	hypothetical protein [Shigella phage Sf13]	100%	8.00E-57	96%	ATE85807.1		
95	64859..65170	-	hypothetical protein [Citrobacter phage Moogle]	97%	4.00E-59	91%	YP_009145717.1		
96	65192..65542	-	major capsid protein [Shigella phage Sf13]	100%	4.00E-68	89%	ATE85809.1	Phage_cap_	pfam03864
97	65717..66334	-	major capsid protein [Shigella phage Sf18]	58%	1.00E-71	94%	ATE86394.1	Phage_cap_	pfam03864
98	66347..67174	-	head maturation protease [Citrobacter phage Michonne]	100%	0	95%	YP_009177327.1	S49_Sppa_	cd07022
99	67306..67497	+	hypothetical protein [Escherichia phage wV8]	100%	1.00E-40	100%	YP_002922880.1		
100	67776..68045	+	hypothetical protein [Salmonella virus VSe11]	87%	9.00E-52	99%	AUE22370.1		
101	68035..68586	-	hypothetical protein [Shigella phage Sf13]	96%	2.00E-121	98%	ATE85819.1		
102	68663..68842	-	hypothetical protein [Shigella phage Sf13]	100%	2.00E-23	81%	ATE85820.1		
103	68839..69033	-	hypothetical protein [Shigella phage Sf13]	93%	6.00E-33	95%	ATE85820.1		
104	69426..69659	+	hypothetical protein [Escherichia phage SUSP1]	100%	2.00E-46	94%	YP_009199379.1		
105	69653..70021	+	hypothetical protein [Shigella phage Sf15]	94%	2.00E-73	95%	ATE86040.1		
106	70018..70239	+	hypothetical protein [Shigella phage Sf13]	100%	4.00E-43	100%	ATE85822.1		
107	70286..70630	+	hypothetical protein [Shigella phage Sf18]	99%	9.00E-71	89%	ATE86380.1		
108	70627..70881	+	tail protein [Shigella phage Sf16]	97%	1.00E-50	95%	ATE86165.1		
109	70888..71136	+	tail protein [Shigella phage Sf13]	48%	6.00E-13	78%	ATE85824.1		
110	71164..71382	+	tail protein [Shigella phage Sf13]	44%	1.00E-05	75%	ATE85824.1		
111	71400..71615	+	lysozyme [Shigella phage Sf15]	97%	2.00E-33	87%	ATE86043.1	RrrD	COG3772
112	71905..72303	+	hypothetical protein [Shigella phage Sf18]	100%	2.00E-81	86%	ATE86377.1		

113	72296..72625	+	hypothetical protein [Shigella phage Sf13]	82%	1.00E-42	78%	ATE85827.1		
114	72671..72964	+	hypothetical protein [Citrobacter phage Moogle]	100%	1.00E-59	95%	YP_009145737.1		
115	72957..73301	+	hypothetical protein [Citrobacter phage Michonne]	100%	9.00E-61	82%	YP_009177347.1		
116	73303..73461	+	hypothetical protein [Shigella phage Sf15]	92%	2.00E-16	75%	ATE86048.1		
117	73445..73783	+	hypothetical protein [Shigella phage Sf13]	90%	9.00E-56	83%	ATE85830.1		
118	73969..74358	+	hypothetical protein [Shigella phage Sf16]	96%	2.00E-65	75%	ATE86158.1		
119	74441..74635	+	hypothetical protein [Shigella phage Sf15]	100%	4.00E-37	98%	ATE86049.1		
120	74640..74813	+	hypothetical protein [Shigella phage Sf13]	94%	1.00E-25	83%	ATE85832.1		
121	74810..75196	+	hypothetical protein [Shigella phage Sf15]	99%	2.00E-70	82%	ATE86156.1		
122	75379..75609	+	hypothetical protein [Escherichia phage EC6]	73%	1.00E-20	77%	YP_009151248.1	Pathogen_b	pfam09593
123	75653..76096	+	hypothetical protein [Shigella phage Sf15]	100%	1.00E-102	100%	ATE86054.1		
124	76201..76395	+	hypothetical protein [Shigella phage Sf13]	100%	8.00E-32	88%	ATE85836.1		
125	76382..76846	+	hypothetical protein [Shigella phage Sf15]	100%	1.00E-111	100%	ATE86056.1		
126	76849..77214	+	hypothetical protein [Shigella phage Sf14]	93%	7.00E-51	78%	AUV62746.1		
127	79530..79652	-	hypothetical protein [Shigella phage Sf13]	100%	1.00E-49	93%	ATE85839.1		
128	79824..80036	-	hypothetical protein [Citrobacter phage Moogle]	100%	4.00E-40	96%	YP_009145747.1		
129	80102..80629	-	hypothetical protein [Escherichia phage SUSP1]	100%	6.00E-74	63%	YP_009199360.1		
130	80829..81011	-	hypothetical protein [Enterobacteria phage vB_EcoM_IME338]	56%	1.00E-09	85%	AWD91315.1		
131	81032..81373	-	hypothetical protein [Citrobacter phage Moogle]	100%	4.00E-80	99%	YP_009145749.1		
132	81459..81656	-	hypothetical protein [Shigella phage Sf13]	100%	2.00E-40	100%	ATE85844.1		
133	81748..82284	-	hypothetical protein [Shigella phage Sf13]	55%	2.00E-62	99%	ATE85845.1		
134	82385..83506	-	putative rIIb protein [Escherichia phage SUSP1]	100%	0	99%	YP_009199333.1		
135	83596..85887	-	rIIA protein [Escherichia phage vB_EcoM_AYO145A]	100%	0	100%	YP_009200740.1	HtpG	COG0326

The predicted ORFs of phage **vB_SdyM_006** , their putative function and consereved domain

	CDS_POSITION	Strand	aa	PI	Mw (Kda)	Best BLAST hit (organism)	Covera	E value	Identity (%)		Consered domain	
1	2046....2258	-	70	4.97	8.2	hypothetical protein [Proteus phage phiP4-3]	100%	3.00E-41	99%	AUM58625.1		
2	2483...4306	-	607	6.39	68.4	DNA topoisomerase subunit [Morganella phage vB_	99%	0	70%	YP_009279861.1	gyrB	TIGR01059
3	4321...4659	-	112	6.9	13.8	hypothetical protein [Proteus phage phiP4-3]	100%	6.00E-78	100%	AUM58621.1		
4	4708...5100	-	130	5.19	15.3	hypothetical protein [Proteus phage phiP4-3]	100%	2.00E-89	100%	AUM58620.1		
5	5103...5531	-	142	4.71	16.6	hypothetical protein [Proteus phage phiP4-3]	100%	5.00E-98	100%	AUM58619.1		
6	5534...5947	-	137	5	15.7	hypothetical protein [Proteus phage PM2]	99%	2.00E-23	41%	ASZ76532.1		
7	5944...6885	-	313	4.64	35.7	hypothetical protein [Proteus phage PM2]	100%	1.00E-122	60%	ASZ76533.1		
8	6890...7105	-	70	5.19	8.2	hypothetical protein [Proteus phage PM2]	94%	3.00E-26	71%	ASZ76534.1		
9	7181...7861	-	226	5.12	26.1	exonuclease A [Proteus phage phiP4-3]	100%	3.00E-164	99%	AUM58615.1	DUF5051	pfam16473
10	7861...9183	-	440	6.44	50.8	DNA helicase [Proteus phage PM2]	100%	0	93%	ASZ76536.1	TraA_Ti	TIGR02768
11	9430...10089	-	219	9.87	25.3	anti-sigma factor [Proteus phage phiP4-3]	100%	1.00E-149	96%	AUM58613.1		
12	10146...10340	-	64	9.16	7.4	hypothetical protein [Proteus phage phiP4-3]	100%	2.00E-37	100%	AUM58612.1	ICE_PFG	TIGR03764
13	10340...10864	-	174	4.86	20.1	dCTP pyrophosphatase [Proteus phage phiP4-3]	100%	3.00E-127	100%	AUM58611.1	dUTPase_	pfam08761
14	10873...11436	-	187	4.89	22.0	hypothetical protein [Proteus phage phiP4-3]	100%	2.00E-133	97%	AUM58610.1		
15	11436...11651	-	70	8.76	8.1	hypothetical protein [Proteus phage phiP4-3]	100%	9.00E-45	100%	AUM58609.1		
16	11644...12201	-	185	4.93	21.2	hypothetical protein [Proteus phage phiP4-3]	100%	3.00E-31	42%	AUM58608.1		
17	12201...12776	-	191	4.59	21.8	hypothetical protein [Proteus phage vB_PmiM_Pm5	95%	8.00E-16	30%	YP_009195438.1	MIB_HE	pfam06701
18	12787...13239	-	150	5.11	17.0	hypothetical protein [Proteus phage phiP4-3]	98%	2.00E-76	80%	AUM58607.1		
19	13239...13424	-	61	4.17	7.0	hypothetical protein [Proteus phage vB_PmiM_Pm5	98%	1.00E-18	77%	YP_009195442.1		
20	13411...13740	-	109	4.81	12.7	hypothetical protein [Proteus phage vB_PmiM_Pm5	97%	6.00E-34	60%	YP_009195443.1		
21	13743...14762	-	339	8.2	39.4	DNA primase subunit [Proteus phage vB_PmiM_Pn	100%	0	99%	YP_009195444.1	DnaG	COG0358
22	14810...15181	-	123	6.3	13.9	hypothetical protein [Proteus phage phiP4-3]	100%	1.00E-84	100%	AUM58602.1		
23	15178...16599	-	473	5.62	53.2	DNA primase/helicase [Proteus phage PM2]	100%	0	96%	ASZ76292.1	phage_Dı	TIGR03600
24	16599...16922	-	107	4.87	12.5	head vertex assembly chaperone [Proteus phage phi	100%	1.00E-69	100%	AUM58600.1	Phage_he	pfam11113
25	16909...18288	-	459	9.26	53.2	putative Hef-like homing endonuclease [Proteus pha	100%	0	83%	ASZ76294.1		
26	18285...19436	-	383	5.51	42.9	RecA-like recombinase protein [Proteus phage phiP	100%	0	99%	AUM58598.1	recA	PRK09519
27	19491...20405	-	304	5.1	36.1	hypothetical protein [Proteus phage PM2]	100%	0	87%	ASZ76299.1		
28	20416...21108	-	230	5.04	26.9	hypothetical protein [Proteus phage phiP4-3]	100%	1.00E-171	100%	AUM58596.1	TS_Pyrin	cd00351
29	21105...21479	-	124	9.3	14.3	hypothetical protein [Proteus phage PM2]	90%	2.00E-39	62%	ASZ76301.1		
30	21479...21664	-	61	9.46	7.0	hypothetical protein [Proteus phage phiP4-3]	100%	7.00E-36	100%	AUM58594.1		
31	21848...23014	-	388	6.24	45.2	peptidase [Proteus phage phiP4-3]	100%	0	100%	AUM58592.1	PrtC	COG0826
32	23053...23259	-	68	9.52	7.4	hypothetical protein [Proteus phage phiP4-3]	100%	2.00E-37	99%	AUM58591.1		
33	23256...23819	-	187	5.72	21.7	hypothetical protein [Proteus phage vB_PmiM_Pm5	99%	2.00E-103	76%	YP_009195458.1	Tmk	COG0125
34	23816...24193	-	125	5.91	14.4	hypothetical protein [Proteus phage phiP4-3]	100%	8.00E-88	99%	AUM58589.1		

35	24190...25839	-	549	6.23	63.1	hypothetical protein [Proteus phage phiP4-3]	100%	0	99%	AUM58588.1		
36	25887...26519	-	210	6.64	23.0	hypothetical protein [Escherichia phage vB_EcoM_	98%	2.00E-104	71%	YP_009037454.1	SIS	pfam01380
37	26520...26969	-	149	9.39	17.1	hypothetical protein [Proteus phage phiP4-3]	99%	2.00E-105	100%	AUM58586.1	TBCC_N	pfam16752
38	27025...29736	-	903	5.99	104.6	DNA polymerase [Proteus phage phiP4-3]	100%	0	99%	AUM58585.1	POLBc	cd00145
39	29791...29988	-	65	6.13	7.7	hypothetical protein [Proteus phage phiP4-3]	100%	1.00E-39	97%	AUM58584.1		
40	29990...30355	-	121	9.06	14.4	translational repressor protein [Proteus phage vB_Pr	100%	1.00E-78	96%	YP_009195464.1	Translat_	pfam01818
41	30358...30930	-	190	5.76	22.4	DNA polymerase accessory protein clamp loader su	100%	6.00E-138	100%	AUM58582.1	Phage_cl	pfam16790
42	30923...31885	-	320	8.14	36.1	DNA polymerase accessory protein clamp loader su	100%	0	100%	AUM58581.1	RarA	COG2256
43	31900...32580	-	226	4.91	25.2	sliding clamp [Proteus phage vB_PmiM_Pm5461]	100%	4.00E-156	95%	YP_009195467.1	gp45-slid	pfam09116
44	32590...32904	-	104	9.55	12.1	RNA polymerase binding protein [Proteus phage ph	100%	1.00E-67	100%	AUM58579.1	Phage_R	pfam10789
45	32897...33079	-	60	4.75	7.4	hypothetical protein [Proteus phage phiP4-3]	100%	1.00E-36	100%	AUM58578.1		
46	33076...34761	-	560	7.15	63.9	recombination endonuclease subunit [Proteus phage	100%	0	100%	AUM58577.1	46	PHA02562
47	34761...35780	-	339	5.04	39.0	recombination endonuclease subunit [Morganella ph	98%	8.00E-172	68%	YP_009279901.1	sbcd	TIGR00619
48	35795...35980	-	61	9.74	7.2	hypothetical protein [Proteus phage phiP4-3]	100%	2.00E-36	100%	AUM58575.1		
49	35958...36287	-	109	8.52	12.9	hypothetical protein [Proteus phage phiP4-3]	100%	4.00E-70	100%	AUM58574.1	DUF2654	pfam10849
50	36280...36483	-	67	3.91	8.2	hypothetical protein PS2_065 [Serratia phage PS2]	73%	1.00E-08	53%	YP_009030112.1		
51	36476...37024	-	182	4.86	21.3	sigma factor for late transcription [Proteus phage ph	97%	1.00E-113	96%	AUM58573.1	55	PHA02547
52	37236...38657	-	473	8.54	54.5	hypothetical protein [Proteus phage phiP4-3]	100%	0	99%	AUM58572.1		
53	38657...38962	-	101	9.48	11.7	hypothetical protein [Proteus phage phiP4-3]	100%	3.00E-67	100%	AUM58571.1	PHA0210	PHA02100
54	38955...39233	-	92	5.29	10.6	hypothetical protein [Proteus phage PM2]	100%	6.00E-49	82%	ASZ76324.1	arsC_rela	TIGR01617
55	39230...39466	-	78	4.94	9.3	hypothetical protein [Proteus phage phiP4-3]	100%	5.00E-51	100%	AUM58569.1		
56	39468...39710	-	80	4.09	9.4	hypothetical protein [Proteus phage phiP4-3]	100%	2.00E-51	100%	AUM58568.1		
57	39719...40195	-	158	5.49	18.3	anaerobic NTP reductase small subunit [Proteus pha	100%	2.00E-113	100%	AUM58567.1	nrdG	PRK11121
58	40234...40566	-	110	9.28	12.8	hypothetical protein [Proteus phage phiP4-3]	100%	2.00E-73	97%	AUM58566.1		
59	40576...40839	-	87	5.15	10.0	hypothetical protein [Proteus phage phiP4-3]	100%	3.00E-57	99%	AUM58565.1		
60	40850...41041	-	63	4.78	7.6	hypothetical protein [Proteus phage phiP4-3]	100%	2.00E-36	98%	AUM58564.1		
61	41085...42956	-	623	5.80	70.2	anaerobic NTP reductase large subunit [Proteus pha	100%	0	99%	AUM58563.1	RNR_III	cd01675
62	42953...43423	-	156	9.1	18.2	recombinase endonuclease VII [Proteus phage phiP4	100%	5.00E-110	99%	AUM58562.1	Endonucl	pfam02945
63	43463...43717	-	84	9.79	9.8	hypothetical protein [Proteus phage phiP4-3]	100%	4.00E-55	100%	AUM58561.1		
64	43866...44138	-	90	7.74	10.5	thioredoxin [Proteus phage PM2]	100%	1.00E-54	90%	ASZ76332.1		
65	44135...44404	-	89	4.54	10.2	hypothetical protein [Proteus phage phiP4-3]	100%	2.00E-58	99%	AUM58558.1		
66	44653...44832	-	59	9.02	7.1	hypothetical protein [Proteus phage phiP4-3]	100%	4.00E-37	100%	AUM58557.1		
67	44835...45830	-	331	8.29	37.7	hypothetical protein [Escherichia phage phiE142]	98%	2.00E-168	71%	ALY07921.1	Nuc-trans	pfam10127
68	45833...46306	-	157	5.91	18.3	hypothetical protein [Proteus phage phiP4-3]	100%	2.00E-111	100%	AUM58555.1		
69	46316...46522	-	68	5.06	8.0	putative fiber protein [Proteus phage phiP4-3]	100%	4.00E-41	100%	AUM58554.1		
70	46519...46740	-	73	7.86	8.6	hypothetical protein [Proteus phage phiP4-3]	100%	2.00E-45	100%	AUM58553.1	PRK0768	PRK07681
71	46734...47312	-	192	6.6	21.7	unkown						
72	47506...48228	-	240	8.78	27.3	hypothetical protein [Proteus phage phiP4-3]	100%	4.00E-174	100%	AUM58550.1		
73	48228...48446	-	72	8.03	8.5	hypothetical protein [Proteus phage PM2]	100%	1.00E-31	72%	ASZ76343.1		

74	48430...48708	-		6.23	10.4	unkown						
75	48709...48915	-	68	9.04	8.0	putative membrane protein [Proteus phage phiP4-3]	100%	8.00E-41	100%	AUM58548.1	Imm_sup	pfam14373
76	48915...49109	-	64	4.93	7.3	putative membrane protein [Proteus phage vB_PmiM	98%	1.00E-19	68%	YP_009195498.1		
77	49111...49296	-	61	6.02	7.3	hypothetical protein [Proteus phage phiP4-3]	100%	2.00E-36	100%	AUM58546.1		
78	49423...49686	-	87	5.28	10.0	hypothetical protein [Proteus phage vB_PmiM_Pm5	100%	3.00E-55	94%	YP_009195501.1	C4_traR_	TIGR02419
79	49687...50001	-	104	6.5	11.9	hypothetical protein [Proteus phage phiP4-3]	100%	2.00E-70	100%	AUM58543.1	DUF2591	pfam10765
80	49998...50570	-	190	6.7	22.4	hypothetical protein [Proteus phage phiP4-3]	100%	2.00E-139	99%	AUM58542.1		
81	50574...50738	-	54	9.45	6.2	hypothetical protein [Proteus phage vB_PmiM_Pm5	98%	2.00E-20	70%	YP_009195503.1		
82	50804...51226	-	140	4.58	16.1	hypothetical protein [Proteus phage phiP4-3]	100%	3.00E-96	99%	AUM58540.1		
83	51228...51839	-	203	5.06	23.5	hypothetical protein [Proteus phage phiP4-3]	100%	4.00E-150	100%	AUM58539.1		
84	51940...52230	-	96	4.77	11.0	hypothetical protein [Klebsiella phage JD18]	100%	7.00E-23	51%	YP_009190674.1	rI.-1	PHA02604
85	52307...52609	-	100	4.84	11.5	lysis inhibition regulator [Proteus phage phiP4-3]	100%	7.00E-68	100%	AUM58537.1	PHA0205	PHA02054
86	52637...52849	-	70	9.3	8.4	hypothetical protein [Proteus phage phiP4-3]	100%	2.00E-41	97%	AUM58536.1		
87	52871...53437	-	188	8.54	21.1	thymidine kinase [Proteus phage vB_PmiM_Pm546	100%	8.00E-103	79%	YP_009195509.1	Tdk	COG1435
88	53439...53651	-	70	9.34	8.3	hypothetical protein [Proteus phage phiP4-3]	100%	6.00E-43	100%	AUM58534.1		
89	53768...54208	-	146	9.17	16.6	hypothetical protein [Proteus phage phiP4-3]	100%	7.00E-104	100%	AUM58533.1		
90	54205...54519	-	104	5.33	11.6	tRNA synthetase modifier [Morganella phage vB_M	81%	8.00E-27	56%	YP_009279952.1		
91	54523...55083	-	186	8.73	21.6	hypothetical protein [Proteus phage vB_PmiM_Pm5	100%	8.00E-124	90%	YP_009195515.1	SLT	pfam01464
92	55080...55499	-	139	9.14	15.9	site-specific RNA endonuclease [Morganella phage	99%	3.00E-32	44%	YP_009279954.1	REGB_T	pfam10715
93	55499...55897	-	132	9.23	15.7	hypothetical protein [Proteus phage PM2]	99%	2.00E-81	87%	ASZ76363.1	Pyr_excis	pfam03013
94	55950...56318	-	122	4.97	13.9	hypothetical protein [Proteus phage vB_PmiM_Pm5	100%	2.00E-80	93%	YP_009195518.1		
95	56318...56482	-	54	8.9	6.3	hypothetical protein [Proteus phage phiP4-3]	100%	4.00E-31	100%	AUM58528.1		
96	56485...56973	-	162	9.17	18.9	hypothetical protein [Proteus phage phiP4-3]	100%	2.00E-117	98%	AUM58526.1		
97	57165...57347	-	60	4.16	7.0	hypothetical protein [Proteus phage phiP4-3]	100%	2.00E-31	88%	AUM58524.1		
98	57349...57720	-	123	9.36	14.1	endolysin [Proteus phage vB_PmiM_Pm5461]	98%	9.00E-71	83%	YP_009195522.1	Peptidase	pfam13539
99	57760...58209	-	149	4.44	17.1	hypothetical protein [Proteus phage phiP4-3]	100%	2.00E-102	100%	AUM58522.1		
100	58199...58450	-	83	5.39	9.5	unkown						
101	58447...58740	-	97	4.75	11.2	hypothetical protein [Proteus phage phiP4-3]	49%	2.00E-26	98%	AUM58520.1		
102	58737...59147	-	136	4.85	15.7	nudix hydrolase [Proteus phage PM2]	98%	5.00E-79	84%	ASZ76370.1	Nudix hydrolase is a s	
103	59156...59377	-	73	4.33	8.6	hypothetical protein [Proteus phage phiP4-3]	100%	1.00E-43	96%	AUM58518.1		
104	59368...59637	-	89	5.16	10.4	hypothetical protein [Proteus phage phiP4-3]	100%	4.00E-57	99%	AUM58517.1		
105	59634...59897	-	87	6.23	10.3	putative membrane protein [Proteus phage phiP4-3]	100%	4.00E-46	83%	AUM58516.1		
106	59897...60163	-	88	9.23	10.9	hypothetical protein [Proteus phage phiP4-3]	100%	3.00E-46	80%	AUM58515.1		
107	60160...60639	-	159	4.78	18.4	hypothetical protein [Proteus phage phiP4-3]	100%	9.00E-97	89%	AUM58514.1		
108	60636...61127	-	163	5.42	19.3	hypothetical protein [Proteus phage phiP4-3]	100%	6.00E-111	96%	AUM58513.1		
109	61178...61774	-	198	5.81	22.3	hypothetical protein [Proteus phage vB_PmiM_Pm5	100%	9.00E-122	90%	YP_009195527.1		
110	61771...62025	-	84	8.72	9.6	hypothetical protein [Proteus phage phiP4-3]	100%	6.00E-51	96%	AUM58511.1		
111	62032...62487	-	151	7.65	17.9	hypothetical protein [Proteus phage phiP4-3]	85%	2.00E-11	30%	AUM58509.1		
112	62477...62914	-	145	7.64	17.3	hypothetical protein [Proteus phage phiP4-3]	100%	1.00E-89	89%	AUM58509.1		

113	62904...63374	-	156	7.62	18.3	hypothetical protein [Proteus phage phiP4-3]	100%	1.00E-90	83%	AUM58508.1		
114	63374...63664	-	96	7.74	10.7	hypothetical protein [Proteus phage vB_PmiM_Pm5	100%	8.00E-63	94%	YP_009195532.1		
115	63762...64112	-	116	4.79	13.5	hypothetical protein [Proteus phage vB_PmiM_Pm5	100%	8.00E-44	58%	YP_009195534.1		
116	64231...64584	-	117	4.73	13.0	hypothetical protein [Proteus phage vB_PmiM_Pm5	100%	4.00E-49	68%	YP_009195536.1	NlpD	COG0739
117	64594...64986	-	130	6.82	13.8	hypothetical protein [Proteus phage PM2]	100%	9.00E-58	67%	ASZ76374.1		
118	65310...65627	-	105	4.25	12.3	hypothetical protein [Proteus phage PM2]	100%	4.00E-47	75%	ASZ76375.1		
119	65980...66585	-	201	10.05	23.5	putative endonuclease segD [Escherichia phage AR	97%	2.00E-53	51%	YP_009167951.1	GIY-YIG	cd10444
120	66597...66779	-	60	8.93	6.6	putative membrane protein [Proteus phage phiP4-3]	100%	8.00E-34	100%	AUM58501.1		
121	67090...67380	-	96	4.52	11.5	hypothetical protein [Proteus phage vB_PmiM_Pm5	82%	7.00E-27	63%	YP_009195540.1		
122	67557...67859	-	100	4.59	11.3	hypothetical protein [Morganella phage vB_MmoM	91%	9.00E-18	52%	YP_009279987.1		
123	67923...68276	-	117	7.88	13.3	hypothetical protein [Proteus phage phiP4-3]	100%	4.00E-53	75%	AUM58497.1		
124	68417...68791	-	124	4.63	14.6	hypothetical protein [Proteus phage phiP4-3]	100%	2.00E-80	95%	AUM58495.1		
125	68810...69097	-	95	7.84	11.0	hypothetical protein [Proteus phage phiP4-3]	100%	4.00E-61	97%	AUM58494.1		
126	69130...69342	-	70	4.54	8.2	hypothetical protein [Proteus phage phiP4-3]	100%	2.00E-38	91%	AUM58493.1		
127	69343...69774	-	143	4.27	16.6	hypothetical protein [Proteus phage phiP4-3]	100%	6.00E-97	96%	AUM58492.1		
128	69883...70161	-	92	6.3	10.6	hypothetical protein [Proteus phage phiP4-3]	100%	4.00E-55	98%	AUM58491.1		
129	70223...70495	-	90	9.36	10.6	unkown						
130	70556...71002	-	148	5.06	16.7	hypothetical protein [Proteus phage PM2]	100%	1.00E-99	97%	ASZ76385.1	57B	PHA02574
131	70992...71252	-	86	4.11	9.7	hypothetical protein [Proteus phage phiP4-3]	100%	1.00E-51	99%	AUM58488.1		
132	71233...71892	-	219	5.49	25.7	dNMP kinase [Proteus phage vB_PmiM_Pm5461]	100%	3.00E-124	77%	YP_009195550.1	DRE_TIM	cd07940
133	71895...72473	-	192	4.85	21.8	tail completion and sheath stabilizer protein [Proteus	100%	1.00E-131	94%	YP_009195551.1	Phage_T4	pfam06841
134	72665...73474	-	269	10.05	31.0	putative DNA end protector during packaging [Prote	100%	0	95%	ASZ76389.1	2	PHA02577
135	73471...73920	-711	149	9.86	18.1	head completion protein [Proteus phage vB_PmiM_	100%	5.00E-95	89%	YP_009195553.1	Tn7_Tnp	pfam08722
136	73972...74523	+	183	5.15	21.6	baseplate wedge subunit [Morganella phage vB_Mm	100%	1.00E-100	74%	YP_009280001.1	Phage_gp	pfam11246
137	74520...76250	+	576	5.43	64.0	baseplate hub + tail lysozyme [Proteus phage PM2]	100%	0	97%	ASZ76392.1	bacteriop	cd00735
138	76250...78313	+	687	4.91	77.7	hypothetical protein [Shigella phage SP18]	99%	0	59%	YP_003934788.1		
139	78315...80234	+	639	5.05	73.1	baseplate wedge subunit [Morganella phage vB_Mm	100%	0	72%	YP_009280005.1	TIGR022	TIGR02243
140	80234...83341	+	1035	6.22	120.5	baseplate wedge subunit [Proteus phage phiP4-3]	100%	0	98%	AUM58479.1		
141	83341...84348	+	335	4.88	38.4	baseplate wedge tail fiber connector [Morganella ph	99%	0	79%	YP_009280007.1	Phage-Gp	pfam09215
142	84407...85258	+	283	5.91	31.1	baseplate wedge subunit and tail pin [Proteus phage	100%	0	96%	YP_009195560.1	T4_gp9_	pfam07880
143	85255...86991	+	578	5.36	64.2	baseplate wedge subunit and tail pin [Proteus phage	70%	0	94%	YP_009195561.1	T4_gp9_	pfam07880
144	86991...87611	+	206	4.82	22.6	baseplate wedge subunit and tail pin [Proteus phage	100%	7.00E-133	86%	YP_009195563.1	GP11	pfam08677
145	87611...88954	+	447	9.49	48.3	short tail fibers protein [Proteus phage vB_PmiM_P	100%	0	88%	YP_009195564.1	34	PHA02584
146	88964...90700	+	578	4.5	63.9	fibritin protein [Proteus phage PM2]	100%	0	88%	ASZ76406.1		
147	90737...91663	+	308	4.57	34.3	neck protein [Proteus phage phiP4-3]	100%	0	100%	AUM58472.1	13	PHA02554
148	91667...92410	+	247	4.34	28.5	neck protein [Morganella phage vB_MmoM_MP1]	99%	1.00E-128	72%	YP_009280015.1	T4_neck-	pfam11649
149	92496...93281	+	261	5.1	30.6	tail sheath stabilization protein [Proteus phage vB_P	100%	0	95%	YP_009195568.1	T4-gp15_	pfam16724
150	93284...93763	+	159	4.67	17.9	terminase small subunit [Morganella phage vB_Mm	94%	1.00E-78	77%	YP_009280017.1	DNA_Pac	pfam11053
151	93735...95534	+	599	5.29	68.4	terminase large subunit [Morganella phage vB_Mmo	99%	0	84%	YP_009280018.1	Terminas	pfam03237

152	95565...97562	+	665	5.32	72.5	tail sheath protein [Morganella phage vB_MmoM_M	99%	0	77%	YP_009280019.1	Phage_sh	pfam04984
153	97600...98091	+	163	4.97	18.6	tail tube protein [Morganella phage vB_MmoM_MP	100%	1.00E-97	78%	YP_009280020.1	Phage_T4	pfam06841
154	98149...99699	+	516	5.41	60.1	portal vertex of head [Morganella phage vB_MmoM	99%	0	68%	YP_009280021.1	Peptidase	pfam07230
155	99700...99927	+	75	4.56	8.6	prohead core protein [Proteus phage phiP4-3]	100%	2.00E-40	99%	AUM58464.1	67	PHA02608
156	99939...100361	+	140	10.2	16.0	prohead core protein [Proteus phage vB_PmiM_Pm	100%	1.00E-76	91%	YP_009195575.1	68	PHA02586
157	100358...100984	+	208	5.97	22.8	prohead core scaffold protein and protease [Morgan	99%	2.00E-111	79%	YP_009280024.1	Peptidase	pfam03420
158	101017...101811	+	264	4.44	29.6	prohead core scaffold protein [Proteus phage vB_Pr	100%	1.00E-145	86%	YP_009195577.1	22	PHA02557
159	101832...103382	+	516	5.12	56.3	major capsid protein [Morganella phage vB_MmoM	100%	0	81%	YP_009280026.1	Gp23	pfam07068
160	103501...104751	+	416	4.83	46.3	capsid vertex protein [Proteus phage PM2]	100%	0	94%	ASZ76424.1	Gp23	pfam07068
161	104808...105371	-	187	8.59	21.0	hypothetical protein [Proteus phage PM2]	100%	2.00E-103	77%	ASZ76425.1		
162	105563...105808	-	81	8.96	9.4	hypothetical protein [Proteus phage phiP4-3]	100%	5.00E-51	100%	AUM58456.1		
163	105816...106820	-	334	5.12	38.0	RNA ligase [Proteus phage PM2]	99%	0	78%	ASZ76428.1	RNA_lig_	TIGR02307
164	106821...107156	-	111	4.75	13.2	hypothetical protein [Proteus phage phiP4-3]	100%	3.00E-66	89%	AUM58454.1		
165	107166...107450	-	94	9.56	11.3	hypothetical protein [Proteus phage vB_PmiM_Pm5	100%	1.00E-55	89%	YP_009195585.1		
166	107447...107638	-	63	10.24	7.6	hypothetical protein [Proteus phage PM2]	100%	3.00E-33	89%	ASZ76431.1	DUF2774	pfam11242
167	107625...107798	-	57	4.63	6.7	hypothetical protein [Proteus phage vB_PmiM_Pm5	100%	3.00E-28	86%	YP_009195587.1		
168	107921...108262	+	113	10.01	13.1	putative membrane protein [Proteus phage vB_PmiM	78%	9.00E-36	73%	YP_009195589.1		
169	108234...108656	-	140	5.28	16.4	hypothetical protein [Proteus phage phiP4-3]	100%	2.00E-100	99%	AUM58449.1		
170	108657...108854	-	65	3.94	7.5	hypothetical protein [Proteus phage PM2]	96%	7.00E-23	70%	ASZ76435.1		
171	108855...109049	-	64	4.31	7.5	hypothetical protein [Proteus phage phiP4-3]	100%	1.00E-38	100%	AUM58447.1		
172	109046...109879	-	277	4.4	32.4	hypothetical protein [Proteus phage phiP4-3]	100%	0	95%	AUM58446.1		
173	109873...110115	-	80	5.89	9.2	hypothetical protein [Proteus phage PM2]	97%	9.00E-20	46%	ASZ76436.1		
174	110272...110616	+	114	9.32	12.9	hypothetical protein [Proteus phage phiP4-3]	100%	1.00E-79	100%	AUM58444.		
175	110646...111560	-	304	4.83	33.3	baseplate tail tube initiator [Morganella phage vB_M	100%	0	80%	YP_009280045.1	Phage_T4	pfam06841
176	111560...112603	-	347	6	38.0	baseplate tail tube cap [Proteus phage vB_PmiM_Pr	100%	0	95%	YP_009195595.1	T4_tail_c	pfam11091
177	112603...114324	-	573	4.79	63.6	baseplate hub subunit [Proteus phage PM2]	100%	0	81%	ASZ76440.1		
178	114311...114829	-	172	6.17	19.8	baseplate hub distal subunit [Proteus phage vB_Pmi	95%	1.00E-104	91%	YP_009195597.1	Phage_hu	pfam11110
179	114792...115937	-	381	5.17	43.7	baseplate hub subunit [Proteus phage vB_PmiM_Pm	100%	0	96%	YP_009195598.1	Phage-tai	pfam09096
180	115915...116688	-	257	5.93	30.4	baseplate hub subunit [Proteus phage PM2]	100%	3.00E-168	89%	ASZ76443.1	T4_basep	pfam12322
181	116734...117318	+	194	4.38	22.5	baseplate hub subunit [Proteus phage vB_PmiM_Pm	100%	1.00E-111	81%	YP_009195600.1	T4_basep	pfam12322
182	117311...117697	+	128	5.4	14.5	baseplate wedge subunit [Proteus phage PM2]	89%	5.00E-75	97%	ASZ76445.1	COG3628	COG3628
183	117937...118347	+	136	7.7	15.7	recombination, repair and ssDNA binding protein [M	100%	2.00E-66	70%	YP_009280053.1	UvsY	pfam11056
184	118344...118715	+	123	4.95	13.9	hypothetical protein [Proteus phage PM2]	100%	6.00E-58	71%	ASZ76447.1	uvsY.-2	PHA02610
185	118733...118954	-	73	4.53	8.6	hypothetical protein [Proteus phage vB_PmiM_Pm5	100%	5.00E-39	93%	YP_009195604.1	UvsW	pfam11637
186	118948...120453	-	501	8.97	57.0	RNA-DNA and DNA-DNA helicase, ATPase [Prote	99%	0	94%	YP_009195605.1	DEXDc	cd00046
187	120509...121222	+	237	4.58	27.7	inhibitor of prohead protease [Proteus phage vB_Pm	100%	1.00E-116	73%	YP_009195606.1		
188	121232...122056	+	274	4.56	31.2	head outer capsid protein [Proteus phage vB_PmiM_	98%	4.00E-62	46%	YP_009195607.1	halo_surf	TIGR04216
189	122090...122359	-	89	5.5	10.2	hypothetical protein [Proteus phage phiP4-3]	100%	4.00E-57	100%	AUM58429.1		
190	122420...124483	-	687	7.05	77.2	RNA polymerase ADP-ribosylase [Proteus phage ph	100%	0	99%	AUM58428.1	ADPrib_e	pfam03496

191	124564...124773	-	69	4.81	8.1	unkown						
192	124862...125098	-	78	4.52	8.7	hypothetical protein [Proteus phage phiP4-3]	100%	2.00E-47	99%	AUM58426.1		
193	125317...126789	-	490	6.16	55.8	DNA ligase [Proteus phage PM2]	100%	0	89%	ASZ76460.1	dnl1	TIGR00574
194	126799...127434	-	210	5.19	24.2	hypothetical protein [Proteus phage PM2]	100%	1.00E-147	95%	ASZ76461.1	30.2	PHA02597
195	127434...127877	-	147	4.67	16.9	hypothetical protein [Proteus phage vB_PmiM_Pm5	100%	4.00E-14	33%	YP_009195615.1		
196	127885...128337	-	150	5.12	16.9	hypothetical protein [Proteus phage phiP4-3]	100%	1.00E-101	99%	AUM58421.1		
197	128339...128659	-	106	4.82	12.2	hypothetical protein [Proteus phage phiP4-3]	100%	7.00E-73	100%	AUM58420.1		
198	128656...129093	-	145	9.51	16.7	hypothetical protein [Proteus phage vB_PmiM_Pm5	100%	2.00E-80	77%	YP_009195617.1	Phage_30	pfam08010
199	129138...129374	-	78	6.17	9.2	hypothetical protein [Proteus phage phiP4-3]	100%	3.00E-47	99%	AUM58418.1		
200	129624...129869	-	81	9.01	9.3	rIII lysis inhibition accessory protein rapid lysis phe	100%	1.00E-40	81%	YP_009280074.1	PRK0675	PRK06756
201	129943...130260	-	105	5.29	11.3	head assembly cochaperone with GroEL [Proteus ph	99%	3.00E-50	76%	YP_009195622.1	Cpn10	pfam00166
202	130260...130577	-	105	8.69	12.2	tail fiber protein [Salmonella phage STP4-a]	100%	4.00E-18	39%	YP_009126152.1	DUF2693	pfam10902
203	130574...131125	-	183	8.18	20.1	hypothetical protein4 [Yersinia phage fHe-Yen9-01]	98%	4.00E-90	72%	ARB06007.1		
204	131414...131752	-	112	6.1	12.8	hypothetical protein [Proteus phage phiP4-3]	100%	3.00E-76	100%	AUM58411.1		
205	131752...132714	-	320	5.94	35.4	phospho-2-dehydro-3-deoxyheptonate aldolase [Pro	100%	0	86%	YP_009195627.1	DAHP_sy	pfam00793
206	132704...132964	-	86	5.16	10.0	hypothetical protein [Proteus phage vB_PmiM_Pm5	96%	2.00E-43	81%	YP_009195628.1		
207	132957...133163	-	68	6.55	7.8	hypothetical protein [Proteus phage phiP4-3]	100%	5.00E-42	100%	AUM58408.1		
208	133169...133360	-	63	9.1	7.3	hypothetical protein [Proteus phage phiP4-3]	100%	1.00E-37	97%	AUM58407.1		
209	133360...134358	-	332	7.2	39.2	3'-phosphatase, 5'-polynucleotide kinase [Proteus ph	100%	0	99%	AUM58406.1	AAA_33	pfam13671
210	134367...134537	-	56	4.52	6.6	hypothetical protein [Proteus phage phiP4-3]	100%	2.00E-33	100%	AUM58405.1		
211	134534...135247	-	237	6.51	26.9	hypothetical protein [Proteus phage phiP4-3]	100%	2.00E-173	100%	AUM58404.1		
212	135250...135462	-	70	4.65	7.9	unkown						
213	135437...135745	-	102	8.77	12.0	hypothetical protein [Proteus phage vB_PmiM_Pm5	96%	8.00E-54	84%	YP_009195634.1		
214	135742...136095	-	117	8.71	13.4	hypothetical protein [Proteus phage PM2]	100%	5.00E-55	69%	ASZ76482.1		
215	136092...136592	-	166	7.01	18.9	inhibitor of host transcription [Proteus phage PM2]	100%	4.00E-95	85%	ASZ76483.1		
216	136650...137768	-	372	4.88	43.1	RNA ligase 1 and tail fiber attachment catalyst [Prot	100%	0	87%	YP_009195637.1	RNA_lig	TIGR02308
217	137765...138175	-	136	9.08	16.2	endonuclease II [Proteus phage PM2]	100%	3.00E-95	99%	ASZ76485.1	GIY-YIG	cd10436
218	138203...139375	-	390	4.91	45.7	aerobic NDP reductase small subunit [Proteus phage	99%	0	94%	YP_009195639.1	NrdF	COG0208
219	139516...141756	-	746	5.84	84.9	ribonucleotide reductase of class Ia (aerobic , alpha	100%	0	93%	ASZ76487.1	NrdA	COG0209
220	141768...142334	-	188	8.87	21.9	hypothetical protein [Proteus phage vB_PmiM_Pm5	99%	1.00E-105	80%	YP_009195642.1		
221	142342...142560	-	72	9.12	8.4	hypothetical protein [Proteus phage vB_PmiM_Pm5	100%	1.00E-38	85%	YP_009195643.1	DUF4326	pfam14216
222	142788...143135	-	115	4.26	13.7	hypothetical protein [Proteus phage phiP4-3]	100%	3.00E-76	99%	AUM58392.1		
223	143144...143995	-	283	5.77	32.8	thymidylate synthase [Morganella phage vB_MmoM	98%	1.00E-152	71%	YP_009280098.1	Thymidyl	pfam00303
224	143980...144252	-	90	6.56	10.5	hypothetical protein [Proteus phage phiP4-3]	100%	5.00E-58	99%	AUM58388.1		
225	144227...144634	-	135	8.68	15.7	hypothetical protein [Proteus phage phiP4-3]	100%	1.00E-94	99%	AUM58387.1		
226	144627...145028	-	133	7.01	15.6	hypothetical protein Pm5461_226 [Proteus phage vI	100%	3.00E-68	76%	YP_009195648.1		
227	145019...145648	-	209	5.78	23.8	dihydrofolate reductase [Proteus phage phiP4-3]	100%	5.00E-154	100%	AUM58385.1	DHFR	cd00209
228	146656...147552	-	298	4.81	33.3	single-stranded DNA-binding protein [Proteus phag	100%	0	93%	ASZ76497.1	gp32	pfam08804
229	147594...148247	-	217	9.09	25.7	DNA helicase loader [Proteus phage PM2]	100%	2.00E-148	95%	ASZ76498.1	T4_Gp59	pfam08993

230	148252...148521	-	89	4.55	10.1	late promoter transcription accessory protein [Proteu	100%	1.00E-53	90%	YP_009195652.1	Trans_co	pfam16805
231	148496...148765	-	89	4.88	10.4	dsDNA binding protein [Proteus phage vB_PmiM_I	100%	1.00E-55	96%	YP_009195653.1	Phage_Ds	pfam11126
232	148774...149691	-	305	5.95 /	35.7	RNase H [Proteus phage vB_PmiM_Pm5461]	100%	0	90%	YP_009195654.1	RNaseH_	pfam09293
233	149730...153386	+	1218	5.31	136.2	long tail fiber proximal subunit [Proteus phage vB_I	100%	0	94%	YP_009195655.1	34	PHA02584
234	153391...154497	+	368	4.71	41.0	long tail fiber proximal connector [Proteus phage vE	100%	0	83%	YP_009195656.1		
235	154704...155168	+	154	5.2	17.1	long tail fiber distal connector [Proteus phage vB_P	100%	2.00E-76	75%	YP_009195657.1	Phage_T4	pfam03903
236	155168...157261	+	697	8.97	76.5	long tail fiber distal subunit [Proteus phage phiP4-3]	100%	0	72%	AUM58374.1	Peptidase	pfam13884
237	157261...157854	+	197	9.33	21.8	distal long tail fiber assembly catalyst [Proteus phag	100%	9.00E-142	99%	AUM58373.1	GP38	pfam05268
238	157896...158519	+	207	5.87	24.2	holin lysis mediator [Proteus phage vB_PmiM_Pm5	100%	5.00E-149	97%	YP_009195660.1	Phage_ho	pfam11031
239	158522...158917	-	131	8.93	14.9	hypothetical protein [Proteus phage vB_PmiM_Pm5	100%	1.00E-47	59%	YP_009195661.1		
240	158920...159174	-	84	5.27	9.6	hypothetical protein [Proteus phage vB_PmiM_Pm5	100%	4.00E-35	69%	YP_009195662.1		
241	159174...159533	-	119	4.3	13.9	hypothetical protein [Proteus phage vB_PmiM_Pm5	100%	1.00E-51	69%	YP_009195663.1		
242	159530...159877	-	115	4.64	12.6	hypothetical protein [Proteus phage phiP4-3]	100%	6.00E-70	94%	AUM58368.1		
243	159879...160151	-	90	5.14	10.5	anti-sigma 70 protein [Proteus phage vB_PmiM_Pm	100%	1.00E-48	89%	YP_009195665.1	AsiA	pfam09010
244	160126...160374	-	82	6.03	9.4	hypothetical protein [Proteus phage phiP4-3]	100%	6.00E-56	100%	AUM58366.1		
245	160381...161094	-	237	7.86	26.8	putative transcriptional regulator [Proteus phage PM	84%	4.00E-126	88%	ASZ76515.1		
246	161113...161550	-	145	8.55	17.3	hypothetical protein [Proteus phage vB_PmiM_Pm5	100%	2.00E-89	86%	YP_009195668.1	PHA018(	PHA01806
247	161547...161738	-	63	4.98	7.7	hypothetical protein [Proteus phage phiP4-3]	100%	2.00E-38	100%	AUM58361.1	PLN0253	PLN02532
248	161731...161976	-	81	4.56	9.1	hypothetical protein [Proteus phage phiP4-3]	100%	4.00E-53	100%	AUM58364.1		
249	162062...163417	-	451	7.63	51.5	DNA topoisomerase [Proteus phage PM2]	100%	0	86%	ASZ76519.1	GyrA	COG0188
250	163472...163945	-	157	9.54	18.0	nucleoid disruption protein [Proteus phage vB_PmiN	98%	4.00E-74	69%	YP_009195672.1		
251	163985...164605	-	206	6.59	23.4	DNA endonuclease IV [Proteus phage vB_PmiM_P	100%	1.00E-131	83%	YP_009195673.1		
252	164658...164858	-	66	9.33	7.4	hypothetical protein [Proteus phage phiP4-3]	100%	9.00E-40	100%	AUM58628.1		

The predicted ORFs of phage **vB_SsoS_008** , their putative function and consereved domain

	POSITION	Strand	aa	PI	Mw (Kda)	Best BLAST hit (organism)	Coverage	E value	Identity	.	Consered domain	
1	98..469	-	123	5.65	13.6	hypothetical protein [Shigella phage phi2457T]	100%	2.00E-74	92%	AYP69390.1		
2	855..1034	-	59	5.55	6.8	hypothetical protein [Shigella phage Sfin-1]	62%	5.00E-07	67%	ATN48438.1		
3	1162..1716	-	184	9.51	20.4	putative site specific DNA methylase [PHAGE_Shigel_Shf	63%	2.00E-74	95%	AYP69365.1		
4	2521..2787	+	88	4.59	10.2	hypothetical protein [Shigella phage phi2457T]	98%	2.00E-46	89%	AYP69373.1		
5	3336..3581	+	81	9.39	9.6	hypothetical protein [Shigella phage phi2457T]	72%	5.00E-29	85%	AYP69378.1		
6	4098..4268	+	56	6.81	6.4	hypothetical protein [Escherichia phage vB_EcoS_SH2]	96%	1.00E-17	72%	ARW57192.1		
7	4553..5020	+	155	4.61	18.1	hypothetical protein [Shigella virus Shfl1]	43%	2.00E-40	97%	YP_004414828.1		
8	5064..5384	+	106	6.89	11.9	hypothetical protein [Shigella phage phi2457T]	71%	8.00E-37	86%	AYP69374.1		
9	5814..6056	+	80	7.96	8.8	hypothetical protein [Shigella phage Sfin-1]	88%	7.00E-27	75%	ATN48426.1		
10	6061..6492	+	143	6.22	16.4	hypothetical protein [Shigella phage phi2457T]	47%	6.00E-44	100%	AYP69414.1		
11	6476..6763	+	95	6.27	11.2	hypothetical protein [Shigella phage phi2457T]	100%	5.00E-62	96%	AYP69396.1	DUF3850	pfam12961
12	6760..6924	+	54	6.7	6.1	hypothetical protein [Shigella phage Sfin-1]	75%	1.00E-19	98%	ATN48422.1		
13	6892..7188	+	98	9.83	10.7	unkown						
14	7210..7371	+	53	9.9	6.0	hypothetical protein[Shigella phage phi2457T]	86%	7.00E-15	80%	AYP69368.1		
15	7870..8046	+	58	8.18	6.5	hypothetical protein[Shigella phage phi2457T]	96%	1.00E-20	79%	AYP69380.1		
16	8068..8238	+	56	3.91	6.5	hypothetical protein[Shigella phage phi2457T]	100%	1.00E-29	93%	AYP69380.1		
17	8333..8878	+	181	5.54	20.7	3'-phosphatase, 5'-polynucleotide kinase [Shigella phage ph	97%	9.00E-94	80%	AYP69371.1	HAD_PNKP-C	cd07502
18	9313..9525	+	70	10.5	8.6	hypothetical protein [Shigella phage phi2457T]	100%	6.00E-19	59%	AYP69370.1		
19	9522..9719	+	65	6.24	7.3	hypothetical protein [Shigella phage Sfin-1]	100%	1.00E-30	82%	ATN48418.1		
20	9753..10001	+	82	4.98	9.6	hypothetical protein [Escherichia virus T1]	84%	2.00E-29	80%	YP_003884.1		
21	9998..10342	+	114	8.44	13.0	hypothetical protein [Shigella phage Sfin-1]	100%	3.00E-56	79%	ATN48416.1		
22	10876..11064	+	62	11.6	7.2	hypothetical protein [Shigella phage phi2457T]	50%	3.00E-08	81%	AYP69423.1		
23	11069..11254	+	61	6.21	7.0	hypothetical protein [Escherichia phage Eco_BIFF]	100%	2.00E-23	79%	AWY08701.1		
24	11544..12071	+	175	9.88	19.9	terminase small subunit [Shigella phage phi2457T]	90%	1.00E-68	73%	AYP69372.1		
25	12226..13902	+	75	9.14	8.9	putative terminase large subunit [Escherichia phage Eco_B	100%	2.00E-87	96%	AWY08628.1	COG5362	COG5362
26	13906..14484	+	192	8.89	21.6	portal protein [Shigella phage SH6]	94%	#######	85%	APC44942.1	phge_rel_HI1409	TIGR01555
27	15009..15284	+	91	4.86	10.4	hypothetical protein [Shigella phage vB_SflS-ISF001]	80%	1.00E-26	77%	ATN94118.1		
28	15335..15577	+	80	5.02	8.9	minor capsid protein [Shigella phage phi2457T]	72%	6.00E-30	100%	AYP69362.1	DUF4297	pfam14130
29	15574..15747	+	57	10.6	6.3	minor capsid protein [Shigella phage Sfin-1]	94%	8.00E-25	91%	ATN48406.1		
30	16032..16232	+	66	8.37	7.2	major capsid protein [Shigella phage SH6]	90%	5.00E-31	95%	APC44972.1		
31	16809..17129	+	106	5.19	11.3	putative major head subunit precursor [Enterobacteria phag	100%	3.00E-58	95%	AWD90853.1		
32	17237..17422	-	61	11	6.7	unkown						
33	17411..17686	+	91	4.94	9.9	hypothetical protein [Escherichia virus T1]	86%	1.00E-42	91%	YP_003896.1		
34	17736..18500	+	254	6.2	26.8	hypothetical protein [Shigella phage phi2457T]	86%	#######	90%	AYP69361.1		
35	18858..19529	+	223	9.32	24.9	hypothetical protein [Escherichia virus T1]	97%	#######	87%	YP_003898.1	COG4834	COG4834

36	19729..20355	+	208	7.06	23.3	hypothetical protein [Escherichia phage JMPW2]	55%	4.00E-63	81%	ALT58155.2	DUF4054	pfam13262
37	20548..20841	+	97	5.43	11.0	hypothetical protein [Escherichia phage ADB-2]	73%	3.00E-30	82%	YP_007112710.1		
38	21090..21380	+	96	9.74	10.4	hypothetical protein [Escherichia phage JMPW1]	63%	3.00E-31	92%	ALT58239.2		
39	21468..21620	+	50	6.29	5.3	hypothetical protein [Shigella virus Shfl1]	52%	0.001	81%	YP_004414857.1		
40	22942..23142	+	66	4.65	7.6	hypothetical protein [Escherichia phage ADB-2]	100%	7.00E-38	97%	YP_007112715.1	DUF1799	pfam08809
41	23184..23441	+	85	7.85	9.4	phage tail tape measure protein [Escherichia phage ADB-2]	94%	2.00E-42	99%	YP_007112716.1		
42	23415..23540	-			0.0	unkown						
43	23561..24400	+	279	8.79	29.3	tail length tape-measure protein [Shigella phage Sfin-1]	97%	#######	92%	ATN48475.1	TMP_2	pfam06791
44	24470..24880	+	136	5	15.0	tail length tape-measure protein [Shigella phage Sfin-1]	100%	2.00E-78	93%	ATN48475.1		
45	25080..26126	+	348	5.5	36.8	putative tail tape measure protein [Escherichia phage Eco_I	100%	0	98%	AWY08643.1	Tape_meas_lam_C	pfam09718
46	26289..26486	+	65	9.62	7.1	tail fibers protein [Shigella phage Sfin-1]	58%	7.00E-18	97%	ATN48474.1	Phage_min_tail	pfam05939
47	26570..27355	+	261	6.82	29.2	minor tail protein [Shigella phage SH6]	100%	0	99%	APC44909.1	phage_tail_L	TIGR01600
48	27352..28092	+	246	5.73	28.5	putative minor tail protein [Escherichia phage Eco_BIFF]	100%	0	99%	AWY08646.1		
49	28089..28688	+	199	8.95	20.9	putative tail assembly protein [Escherichia virus T1]	100%	#######	99%	YP_003911.1	COG4723	COG4723
50	28834..29055	+	73	4.53	8.0	putative tail fiber protein [Escherichia virus T1]	82%	7.00E-30	95%	YP_003912.1	COG4733	COG4733
51	29045..29392	+	115	6.29	13.0	tail fiber protein [Shigella phage vB_SflS-ISF001]	96%	1.00E-65	99%	ATN94099.1	COG4733	COG4733
52	29352..29735	+	127	8.93	14.8	putative tail fiber protein [Escherichia phage Eco_BIFF]	84%	2.00E-66	99%	AWY08648.1	COG4733	COG4733
53	29758..30261	+	167	5.09	19.2	tail fiber protein [Shigella phage vB_SflS-ISF001]	80%	3.00E-87	100%	ATN94099.1	Phage-tail_3	pfam13550
54	30224..31327	+	367	4.99	40.5	putative tail fiber protein [Escherichia phage Eco_BIFF]	82%	0	99%	AWY08648.1	COG4733	COG4733
55	31363..32322	+	319	5.04	34.6	putative tail fiber protein [Escherichia virus T1]	90%	#######	89%	YP_003912.1	DUF1983	pfam09327
56	32627..32851	-	74	4.09	8.0	hypothetical protein [Shigella phage SH6]	75%	5.00E-21	82%	APC44975.1		
57	33169..33390	-	73	9.13	8.1	hypothetical protein [Shigella phage Sfin-1]	45%	1.00E-10	85%	ATN48468.1	Esterase_lipase	cd00312
58	34026..35090	+	354	5.09	40.2	exodeoxyribonuclease VIII [Escherichia virus T1]	100%	0	99%	YP_003916.1	DUF3799	pfam12684
59	35132..35812	+	226	8.49	25.2	putative recombination protein [Escherichia virus T1]	100%	#######	100%	YP_003917.1	ERF	pfam04404
60	35859..36272	+	137	6.11	15.7	hypothetical protein [Shigella phage vB_SsoS-ISF002]	100%	7.00E-96	98%	ASD50899.1	DUF3127	pfam11325
61	36312..37169	-	285	7.71	32.1	putative tail fiber [Escherichia phage Eco_BIFF]	97%	#######	78%	AWY08655.1		
62	37252..38070	-	272	4.45	28.8	tail fibers protein [Shigella phage Sfin-1]	98%	#######	92%	ATN48464.1	PHA00430	PHA00430
63	38171..38662	-	163	4.91	18.6	DNA primase/helicase [Shigella phage phi2457T]	100%	#######	98%	AYP69359.1		
64	38655..39059	-	134	9.56	14.9	DNA primase [Shigella phage Sfin-1]	97%	8.00E-89	96%	ATN48463.1	Prim_Zn_Ribbon	smart00778
65	39237..39614	-	125	10.5	14.1	hypothetical protein [Shigella phage Sfin-1]	100%	8.00E-83	96%	ATN48462.1	P22_Cro	pfam14549
66	39723..41735	+	670	6.13	75.8	putative ATP-dependent helicase [Escherichia phage Eco_I	100%	0	98%	AWY08658.1	Helicase_C	pfam00271
67	41732..42148	+	138	8.59	15.8	hypothetical protein [Shigella phage vB_SsoS-ISF002]	100%	6.00E-98	100%	ASD50894.1	VRR_NUC	pfam08774
68	42312..42950	+	212	8.58	24.2	putative DNA N-6-adenine-methyltransferase [Shigella pha	100%	#######	97%	ASD50893.1	Dam	pfam05869
69	42950..43198	+	82	4.48	9.5	hypothetical protein [Escherichia phage Eco_BIFF]	100%	2.00E-53	98%	AWY08662.1		
70	43240..43473	+	77	9.42	9.0	hypothetical protein [Shigella phage SH6]	96%	3.00E-42	92%	APC44907.1		
71	43451..43723	+	90	9.79	10.5	hypothetical protein [Shigella phage Sfin-1]	100%	8.00E-55	94%	ATN48456.1		
72	44034..44465	+	143	10	16.2	hypothetical protein [Escherichia virus T1]	65%	3.00E-42	81%	YP_003929.1		
73	44663..44857	+	64	7.95	7.4	hypothetical protein [Enterobacteria phage vB_EcoS_IME1	81%	1.00E-21	81%	AWD90901.1	MPP_Mre11_N	cd00840
74	44943..45239	+	98	7.02	11.1	hypothetical protein [Shigella virus Shfl1]	100%	6.00E-55	88%	YP_004414881.1		

75	45270..45446	+	58	6.08	6.6	hypothetical protein [Escherichia virus T1]	100%	1.00E-32	95%	YP_003931.1		
76	45517..45942	+	140	9.97	15.7	endolysin [Shigella phage Sfin-1]	65%	2.00E-54	90%	ATN48451.1	RrrD	COG3772
77	45942..46322	+	126	6.71	13.3	spanin [Shigella phage SH6]	79%	2.00E-50	84%	APC44951.1		
78	46357..46698	-	113	5.46	12.4	hypothetical protein [Escherichia phage Eco_BIFF]	61%	4.00E-34	86%	AWY08670.1		
79	46762..46926	-	54	5.29	6.6	hypothetical protein [Escherichia phage Eco_BIFF]	100%	8.00E-30	96%	AWY08671.1		
80	46996..47169	-	57	9.14	6.8	hypothetical protein [Enterobacteria phage vB_EcoS_IME1	100%	7.00E-30	93%	AWD90895.1		
81	47248..48741	-	497	7.62	55.4	hypothetical protein [Escherichia phage vB_EcoS_SH2]	95%	0	95%	ARW57256.1	DUF3987	pfam13148
82	48755..49186	-	143	9.47	16.7	hypothetical protein [Shigella phage phi2457T]	72%	4.00E-61	91%	AYP69383.1		
83	49307..49603	-	98	4.13	10.8	hypothetical protein [Shigella phage vB_SsoS-ISF002]	54%	2.00E-27	94%	ASD50958.1		
