# Supplementary material for: Biodiversity of New Lytic Bacteriophages Infecting Shigella spp. in Freshwater Environment
Source: Front Microbiol. 2021 Feb 17;12:619323. doi: 10.3389/fmicb.2021.619323 (PMC7925395; doi:10.3389/fmicb.2021.619323)
Supplement: Supplementary file 4 [file Data_Sheet_4.pdf]

CoreGenes analysis of vB_SflM_004

The number of homologs in each column is : 93
Total number of genes in genome 1 : 135
% in common, with respect to genome 1: 68.89
Total number of genes in genome 2 : 140
% in common, with respect to genome 2: 66.43
Total number of genes in genome 3 : 132
% in common, with respect to genome 3: 70.45
Total number of genes in genome 4 : 132
% in common, with respect to genome 4: 70.45
Total number of genes in genome 5 : 130
% in common, with respect to genome 5: 71.54

**Function**	**vB_SflM_004 (MK295205)**	**wV8 (EU877232)**	**phage 11 (KP869109)**	**phage 12 (KP869110)**	**BPS15Q2 (KX405003)**
hypothetical protein	AZV01390.1	ACJ71844.1	AKE46524.1	AKE46639.1	ANT42360.1
hypothetical protein	AZV01395.1	ACJ71840.1	AKE46519.1	AKE46644.1	ANT42357.1
hypothetical protein	AZV01396.1	ACJ71839.1	AKE46518.1	AKE46645.1	ANT42486.1
hypothetical protein	AZV01397.1	ACJ71838.1	AKE46517.1	AKE46646.1	ANT42485.1
hypothetical protein	AZV01400.1	ACJ71836.1	AKE46515.1	AKE46648.1	ANT42483.1
hypothetical protein	AZV01401.1	ACJ71834.1	AKE46513.1	AKE46650.1	ANT42482.1
hypothetical protein	AZV01402.1	ACJ71833.1	AKE46512.1	AKE46651.1	ANT42481.1
prohead assembly scaffo	AZV01404.1	ACJ71832.1	AKE46511.1	AKE46652.1	ANT42480.1
hypothetical protein	AZV01405.1	ACJ71831.1	AKE46510.1	AKE46653.1	ANT42479.1
hypothetical protein	AZV01406.1	ACJ71830.1	AKE46509.1	AKE46654.1	ANT42478.1
tail tube protein	AZV01407.1	ACJ71829.1	AKE46508.1	AKE46655.1	ANT42477.1
tail sheath monomer	AZV01408.1	ACJ71828.1	AKE46507.1	AKE46656.1	ANT42476.1
hypothetical protein	AZV01410.1	ACJ71827.1	AKE46506.1	AKE46657.1	ANT42474.1
hypothetical protein	AZV01411.1	ACJ71826.1	AKE46505.1	AKE46658.1	ANT42473.1
phosphatase	AZV01413.1	ACJ71824.1	AKE46503.1	AKE46660.1	ANT42470.1
hypothetical protein	AZV01414.1	ACJ71823.1	AKE46502.1	AKE46661.1	ANT42469.1
tail assembly protein	AZV01415.1	ACJ71821.1	AKE46500.1	AKE46663.1	ANT42467.1
hypothetical protein	AZV01416.1	ACJ71820.1	AKE46499.1	AKE46664.1	ANT42466.1
o-spanin	AZV01417.1	ACJ71819.1	AKE46498.1	AKE46665.1	ANT42465.2
i-spanin	AZV01418.1	ACJ71818.1	AKE46497.1	AKE46666.1	ANT42464.1
hypothetical protein	AZV01419.1	ACJ71817.1	AKE46496.1	AKE46667.1	ANT42463.1
polynucleotide kinase	AZV01420.1	ACJ71816.1	AKE46495.1	AKE46668.1	ANT42462.1

rIIB protein	AZV01421.1	ACJ71814.1	AKE46493.1	AKE46670.1	ANT42460.1
rIIA lysis inhibitor	AZV01422.1	ACJ71813.1	AKE46492.1	AKE46671.1	ANT42459.1
hypothetical protein	AZV01423.1	ACJ71951.1	AKE46622.1	AKE46673.1	ANT42457.1
nictotinate phosphoribo	AZV01425.1	ACJ71950.1	AKE46621.1	AKE46674.1	ANT42456.1
putative ribose-phospha	AZV01427.1	ACJ71949.1	AKE46620.1	AKE46675.1	ANT42454.1
hypothetical protein	AZV01428.1	ACJ71948.1	AKE46619.1	AKE46676.1	ANT42453.1
hypothetical protein	AZV01429.1	ACJ71947.1	AKE46618.1	AKE46677.1	ANT42452.1
hypothetical protein	AZV01430.1	ACJ71946.1	AKE46617.1	AKE46678.1	ANT42451.1
hypothetical protein	AZV01431.1	ACJ71945.1	AKE46616.1	AKE46679.1	ANT42450.1
anaerobic nucleoside-tri	AZV01432.1	ACJ71943.1	AKE46615.1	AKE46680.1	ANT42448.1
tail tube protein	AZV01433.1	ACJ71941.1	AKE46613.1	AKE46682.1	ANT42446.1
anaerobic ribonucleosid	AZV01436.1	ACJ71938.1	AKE46611.1	AKE46684.1	ANT42445.1
hypothetical protein	AZV01437.1	ACJ71937.1	AKE46610.1	AKE46685.1	ANT42444.1
ribonucleoside triphosph	AZV01438.1	ACJ71935.1	AKE46608.1	AKE46687.1	ANT42442.1
hypothetical protein	AZV01439.1	ACJ71934.1	AKE46607.1	AKE46688.1	ANT42441.1
ribonucleoside triphosph	AZV01440.1	ACJ71933.1	AKE46606.1	AKE46689.1	ANT42440.1
hypothetical protein	AZV01441.1	ACJ71932.1	AKE46605.1	AKE46690.1	ANT42439.1
hypothetical protein	AZV01442.1	ACJ71931.1	AKE46604.1	AKE46691.1	ANT42438.1
phosphoribosyl-ATP pyr	AZV01443.1	ACJ71930.1	AKE46603.1	AKE46692.1	ANT42437.1
hypothetical protein	AZV01444.1	ACJ71929.1	AKE46602.1	AKE46693.1	ANT42436.1
NAD synthetase	AZV01445.1	ACJ71928.1	AKE46601.1	AKE46694.1	ANT42435.1
HNH endonuclease	AZV01446.1	ACJ71927.1	AKE46600.1	AKE46695.1	ANT42434.1
exodeoxyribonuclease	AZV01447.1	ACJ71926.1	AKE46599.1	AKE46696.1	ANT42433.1
hypothetical protein	AZV01448.1	ACJ71925.1	AKE46598.1	AKE46697.1	ANT42432.1
DNA primase/helicase	AZV01449.1	ACJ71922.1	AKE46595.1	AKE46700.1	ANT42430.1
hypothetical protein	AZV01450.1	ACJ71921.1	AKE46594.1	AKE46701.1	ANT42429.1
deoxynucleotide monop	AZV01451.1	ACJ71920.1	AKE46593.1	AKE46702.1	ANT42428.1
minor tail protein	AZV01452.1	ACJ71919.1	AKE46592.1	AKE46703.1	ANT42427.1
hypothetical protein	AZV01453.1	ACJ71918.1	AKE46591.1	AKE46704.1	ANT42426.1
DNA polymerase	AZV01454.1	ACJ71917.1	AKE46590.1	AKE46705.1	ANT42423.1
hypothetical protein	AZV01456.1	ACJ71916.1	AKE46589.1	AKE46706.1	ANT42421.1
hypothetical protein	AZV01457.1	ACJ71913.1	AKE46586.1	AKE46709.1	ANT42418.1
hypothetical protein	AZV01458.1	ACJ71909.1	AKE46582.1	AKE46713.1	ANT42415.1
hypothetical protein	AZV01459.1	ACJ71908.1	AKE46581.1	AKE46714.1	ANT42414.1
hypothetical protein	AZV01460.1	ACJ71907.1	AKE46580.1	AKE46715.1	ANT42413.1
transcriptional regulator	AZV01461.1	ACJ71905.1	AKE46578.1	AKE46717.1	ANT42411.1
nucleoside triphosphate	AZV01462.1	ACJ71904.1	AKE46577.1	AKE46718.1	ANT42410.1
hypothetical protein	AZV01464.1	ACJ71903.1	AKE46576.1	AKE46719.1	ANT42409.1
hypothetical protein	AZV01466.1	ACJ71901.1	AKE46574.1	AKE46721.1	ANT42407.1

thymidylate synthase	AZV01467.1	ACJ71900.1	AKE46573.1	AKE46722.1	ANT42406.1
holin	AZV01468.1	ACJ71899.1	AKE46572.1	AKE46723.1	ANT42405.1
hypothetical protein	AZV01469.1	ACJ71898.1	AKE46571.1	AKE46724.1	ANT42404.1
tail fiber protein	AZV01470.1	ACJ71897.1	AKE46570.1	AKE46725.1	ANT42403.1
tail fiber protein	AZV01474.1	ACJ71896.1	AKE46569.1	AKE46726.1	ANT42402.1
hypothetical protein	AZV01475.1	ACJ71894.1	AKE46567.1	AKE46727.1	ANT42400.1
baseplate assembly prot	AZV01476.1	ACJ71893.1	AKE46566.1	AKE46729.1	ANT42399.1
hypothetical protein	AZV01478.1	ACJ71884.1	AKE46557.1	AKE46738.1	ANT42390.1
hypothetical protein	AZV01479.1	ACJ71883.1	AKE46556.1	AKE46739.1	ANT42389.1
hypothetical protein	AZV01482.1	ACJ71881.1	AKE46554.1	AKE46741.1	ANT42387.1
hypothetical protein	AZV01483.1	ACJ71880.1	AKE46553.1	AKE46742.1	ANT42386.1
hypothetical protein	AZV01484.1	ACJ71879.1	AKE46552.1	AKE46743.1	ANT42385.1
major capsid protein	AZV01486.1	ACJ71876.1	AKE46550.1	AKE46744.1	ANT42383.1
head maturation protea	AZV01487.1	ACJ71875.1	AKE46549.1	AKE46746.1	ANT42382.1
hypothetical protein	AZV01488.1	ACJ71911.1	AKE46584.1	AKE46711.1	ANT42417.1
hypothetical protein	AZV01489.1	ACJ71914.1	AKE46587.1	AKE46708.1	ANT42419.1
hypothetical protein	AZV01490.1	ACJ71859.1	AKE46539.1	AKE46624.1	ANT42373.1
hypothetical protein	AZV01492.1	ACJ71858.1	AKE46538.1	AKE46625.1	ANT42372.1
hypothetical protein	AZV01494.1	ACJ71855.1	AKE46535.1	AKE46628.1	ANT42371.1
hypothetical protein	AZV01496.1	ACJ71854.1	AKE46534.1	AKE46629.1	ANT42370.1
tail protein	AZV01497.1	ACJ71853.1	AKE46533.1	AKE46630.1	ANT42369.1
endolysin	AZV01500.1	ACJ71852.1	AKE46532.1	AKE46631.1	ANT42368.1
hypothetical protein	AZV01501.1	ACJ71851.1	AKE46531.1	AKE46632.1	ANT42367.1
hypothetical protein	AZV01502.1	ACJ71850.1	AKE46530.1	AKE46633.1	ANT42366.1
hypothetical protein	AZV01503.1	ACJ71849.1	AKE46529.1	AKE46634.1	ANT42365.1
hypothetical protein	AZV01504.1	ACJ71848.1	AKE46528.1	AKE46635.1	ANT42364.1
hypothetical protein	AZV01506.1	ACJ71847.1	AKE46527.1	AKE46636.1	ANT42363.1
hypothetical protein	AZV01507.1	ACJ71846.1	AKE46526.1	AKE46637.1	ANT42362.1
hypothetical protein	AZV01509.1	ACJ71845.1	AKE46525.1	AKE46638.1	ANT42361.1
hypothetical protein	AZV01511.1	ACJ71843.1	AKE46522.1	AKE46641.1	ANT42359.1
hypothetical protein	AZV01518.1	ACJ71837.1	AKE46516.1	AKE46647.1	ANT42484.1
hypothetical protein	AZV01522.1	ACJ71815.1	AKE46494.1	AKE46669.1	ANT42461.1

CoreGenes analysis of vB_SdyM_006

The number of homologs in each column is : 212
Total number of genes in genome 1 : 252
% in common, with respect to genome 1: 84.13
Total number of genes in genome 2 : 270
% in common, with respect to genome 2: 78.52
Total number of genes in genome 3 : 256
% in common, with respect to genome 3: 82.81

**Function**	**vB_SdyM_006 (MK295204)**	**phiP4-3 (MG696114)**	**vB_PmiM_Pm5461 (KP890823)**
hypothetical protein	AZV01138.1	AUM58625.1	AKA61864.1
DNA topoisomerase subunit	AZV01139.1	AUM58622.1	AKA61866.1
hypothetical protein	AZV01140.1	AUM58621.1	AKA61870.1
hypothetical protein	AZV01141.1	AUM58620.1	AKA61871.1
hypothetical protein	AZV01143.1	AUM58618.1	AKA61872.1
hypothetical protein	AZV01144.1	AUM58617.1	AKA61874.1
hypothetical protein	AZV01145.1	AUM58616.1	AKA61875.1
exonuclease A	AZV01146.1	AUM58615.1	AKA61876.1
DNA helicase	AZV01147.1	AUM58614.1	AKA61877.1
anti-sigma factor	AZV01148.1	AUM58613.1	AKA61879.1
hypothetical protein	AZV01149.1	AUM58612.1	AKA61880.1
dCTP pyrophosphatase	AZV01150.1	AUM58611.1	AKA61881.1
hypothetical protein	AZV01151.1	AUM58610.1	AKA61882.1
hypothetical protein	AZV01155.1	AUM58607.1	AKA61884.1
hypothetical protein	AZV01156.1	AUM58605.1	AKA61886.1
hypothetical protein	AZV01157.1	AUM58604.1	AKA61887.1
DNA primase subunit	AZV01158.1	AUM58603.1	AKA61888.1
hypothetical protein	AZV01159.1	AUM58602.1	AKA61889.1
DNA primase/helicase	AZV01160.1	AUM58601.1	AKA61890.1
head vertex assembly chaperone	AZV01161.1	AUM58600.1	AKA61891.1
Hef-like homing endonuclease	AZV01162.1	AUM58599.1	AKA61892.1
RecA-like recombinase protein	AZV01163.1	AUM58598.1	AKA61893.1
hypothetical protein	AZV01164.1	AUM58597.1	AKA61896.1
hypothetical protein	AZV01165.1	AUM58596.1	AKA61897.1
hypothetical protein	AZV01166.1	AUM58595.1	AKA61898.1
peptidase	AZV01168.1	AUM58592.1	AKA61900.1
hypothetical protein	AZV01169.1	AUM58591.1	AKA61901.1
hypothetical protein	AZV01170.1	AUM58590.1	AKA61902.1
hypothetical protein	AZV01171.1	AUM58589.1	AKA61903.1
hypothetical protein	AZV01172.1	AUM58588.1	AKA61904.1
hypothetical protein	AZV01174.1	AUM58586.1	AKA61905.1
DNA polymerase	AZV01175.1	AUM58585.1	AKA61906.1
hypothetical protein	AZV01176.1	AUM58584.1	AKA61907.1
translational repressor protein	AZV01177.1	AUM58583.1	AKA61908.1
DNA polymerase accessory protein c	AZV01178.1	AUM58582.1	AKA61909.1
hypothetical protein	AZV01179.1	AUM58581.1	AKA61910.1
sliding clamp	AZV01180.1	AUM58580.1	AKA61911.1

RNA polymerase binding protein	AZV01181.1	AUM58579.1	AKA61912.1
hypothetical protein	AZV01182.1	AUM58578.1	AKA61913.1
hypothetical protein	AZV01183.1	AUM58577.1	AKA61914.1
hypothetical protein	AZV01184.1	AUM58576.1	AKA61915.1
hypothetical protein	AZV01185.1	AUM58575.1	AKA61916.1
hypothetical protein	AZV01186.1	AUM58574.1	AKA61917.1
sigma factor for late transcription	AZV01188.1	AUM58573.1	AKA61918.1
hypothetical protein	AZV01189.1	AUM58572.1	AKA61919.1
hypothetical protein	AZV01190.1	AUM58571.1	AKA61920.1
hypothetical protein	AZV01191.1	AUM58570.1	AKA61921.1
hypothetical protein	AZV01192.1	AUM58569.1	AKA61922.1
anaerobic NTP reductase small subu	AZV01194.1	AUM58567.1	AKA61923.1
hypothetical protein	AZV01195.1	AUM58566.1	AKA61924.1
anaerobic NTP reductase large subu	AZV01198.1	AUM58563.1	AKA61925.1
recombinase endonuclease VII	AZV01199.1	AUM58562.1	AKA61926.1
thioredoxin	AZV01201.1	AUM58559.1	AKA61928.1
hypothetical protein	AZV01202.1	AUM58558.1	AKA61929.1
hypothetical protein	AZV01203.1	AUM58557.1	AKA61931.1
hypothetical protein	AZV01204.1	AUM58396.1	AKA61932.1
hypothetical protein	AZV01205.1	AUM58555.1	AKA61934.1
hypothetical protein	AZV01207.1	AUM58553.1	AKA61935.1
hypothetical protein	AZV01208.1	AUM58552.1	AKA61936.1
hypothetical protein	AZV01209.1	AUM58550.1	AKA61938.1
hypothetical protein	AZV01210.1	AUM58549.1	AKA61939.1
membrane protein	AZV01212.1	AUM58548.1	AKA61941.1
membrane protein	AZV01213.1	AUM58547.1	AKA61942.1
hypothetical protein	AZV01214.1	AUM58546.1	AKA61943.1
hypothetical protein	AZV01215.1	AUM58544.1	AKA61945.1
hypothetical protein	AZV01217.1	AUM58542.1	AKA61946.1
hypothetical protein	AZV01218.1	AUM58541.1	AKA61947.1
hypothetical protein	AZV01219.1	AUM58540.1	AKA61948.1
hypothetical protein	AZV01220.1	AUM58539.1	AKA61949.1
hypothetical protein	AZV01221.1	AUM58538.1	AKA61950.1
lysis inhibition regulator	AZV01222.1	AUM58537.1	AKA61951.1
hypothetical protein	AZV01223.1	AUM58536.1	AKA61952.1
thymidine kinase	AZV01224.1	AUM58535.1	AKA61953.1
hypothetical protein	AZV01225.1	AUM58534.1	AKA61954.1
hypothetical protein	AZV01226.1	AUM58533.1	AKA61957.1
tRNA synthetase modifier	AZV01227.1	AUM58532.1	AKA61958.1
hypothetical protein	AZV01228.1	AUM58531.1	AKA61959.1
site-specific RNA endonuclease	AZV01229.1	AUM58530.1	AKA61960.1
hypothetical protein	AZV01230.1	AUM58529.1	AKA61961.1
hypothetical protein	AZV01231.1	AUM58527.1	AKA61962.1
hypothetical protein	AZV01232.1	AUM58528.1	AKA61963.1
hypothetical protein	AZV01233.1	AUM58526.1	AKA61964.1
hypothetical protein	AZV01235.1	AUM58523.1	AKA61966.1
hypothetical protein	AZV01236.1	AUM58522.1	AKA61967.1
nudix hydrolase	AZV01239.1	AUM58519.1	AKA61968.1
hypothetical protein	AZV01240.1	AUM58518.1	AKA61969.1
membrane protein	AZV01242.1	AUM58516.1	AKA61970.1

hypothetical protein	AZV01246.1	AUM58512.1	AKA61971.1
hypothetical protein	AZV01247.1	AUM58511.1	AKA61972.1
hypothetical protein	AZV01250.1	AUM58508.1	AKA61974.1
hypothetical protein	AZV01251.1	AUM58507.1	AKA61976.1
hypothetical protein	AZV01252.1	AUM58506.1	AKA61978.1
hypothetical protein	AZV01253.1	AUM58505.1	AKA61980.1
hypothetical protein	AZV01254.1	AUM58504.1	AKA61979.1
hypothetical protein	AZV01255.1	AUM58503.1	AKA61981.1
membrane protein	AZV01257.1	AUM58501.1	AKA61983.1
hypothetical protein	AZV01258.1	AUM58500.1	AKA61984.1
hypothetical protein	AZV01260.1	AUM58497.1	AKA61988.1
hypothetical protein	AZV01267.1	AUM58489.1	AKA61992.1
hypothetical protein	AZV01268.1	AUM58488.1	AKA61993.1
dNMP kinase	AZV01269.1	AUM58487.1	AKA61994.1
tail completion and sheath stabilizer	AZV01270.1	AUM58486.1	AKA61995.1
DNA end protector during packaging	AZV01271.1	AUM58485.1	AKA61996.1
head completion protein	AZV01272.1	AUM58484.1	AKA61997.1
baseplate wedge subunit	AZV01273.1	AUM58483.1	AKA61998.1
baseplate hub + tail lysozyme	AZV01274.1	AUM58482.1	AKA61999.1
hypothetical protein	AZV01275.1	AUM58481.1	AKA62000.1
baseplate wedge subunit	AZV01276.1	AUM58480.1	AKA62001.1
baseplate wedge subunit	AZV01277.1	AUM58479.1	AKA62002.1
baseplate wedge tail fiber connector	AZV01278.1	AUM58478.1	AKA62003.1
baseplate wedge subunit and tail pin	AZV01279.1	AUM58477.1	AKA62004.1
baseplate wedge subunit and tail pin	AZV01280.1	AUM58476.1	AKA62005.1
baseplate wedge subunit and tail pin	AZV01281.1	AUM58475.1	AKA62007.1
short tail fibers protein	AZV01282.1	AUM58474.1	AKA62008.1
fibritin protein	AZV01283.1	AUM58473.1	AKA62009.1
neck protein	AZV01284.1	AUM58472.1	AKA62010.1
neck protein	AZV01285.1	AUM58471.1	AKA62011.1
tail sheath stabilization protein	AZV01286.1	AUM58470.1	AKA62012.1
terminase small subunit	AZV01287.1	AUM58469.1	AKA62013.1
terminase large subunit	AZV01288.1	AUM58468.1	AKA62014.1
tail sheath protein	AZV01289.1	AUM58467.1	AKA62015.1
tail tube protein	AZV01290.1	AUM58466.1	AKA62016.1
portal vertex of head	AZV01291.1	AUM58465.1	AKA62017.1
prohead core protein	AZV01292.1	AUM58464.1	AKA62018.1
prohead core protein	AZV01293.1	AUM58463.1	AKA62019.1
prohead core scaffold protein and pr	AZV01294.1	AUM58462.1	AKA62020.1
prohead core scaffold protein	AZV01295.1	AUM58461.1	AKA62021.1
major capsid protein	AZV01296.1	AUM58460.1	AKA62022.1
capsid vertex protein	AZV01297.1	AUM58459.1	AKA62023.1
hypothetical protein	AZV01298.1	AUM58458.1	AKA62024.1
hypothetical protein	AZV01299.1	AUM58456.1	AKA62026.1
RNA ligase	AZV01300.1	AUM58455.1	AKA62027.1
hypothetical protein	AZV01301.1	AUM58454.1	AKA62028.1
hypothetical protein	AZV01302.1	AUM58453.1	AKA62029.1
hypothetical protein	AZV01303.1	AUM58452.1	AKA62030.1
hypothetical protein	AZV01304.1	AUM58451.1	AKA62031.1
hypothetical protein	AZV01306.1	AUM58449.1	AKA62034.1

hypothetical protein	AZV01310.1	AUM58445.1	AKA62035.1
hypothetical protein	AZV01311.1	AUM58444.1	AKA62037.1
baseplate tail tube initiator	AZV01312.1	AUM58443.1	AKA62038.1
baseplate tail tube cap	AZV01313.1	AUM58442.1	AKA62039.1
baseplate hub subunit	AZV01314.1	AUM58441.1	AKA62040.1
baseplate hub distal subunit	AZV01315.1	AUM58440.1	AKA62041.1
baseplate hub subunit	AZV01316.1	AUM58439.1	AKA62042.1
baseplate hub subunit	AZV01317.1	AUM58438.1	AKA62043.1
baseplate hub subunit	AZV01318.1	AUM58437.1	AKA62044.1
baseplate wedge subunit	AZV01319.1	AUM58436.1	AKA62045.1
recombination, repair and ssDNA bir	AZV01320.1	AUM58435.1	AKA62046.1
hypothetical protein	AZV01321.1	AUM58434.1	AKA62047.1
hypothetical protein	AZV01322.1	AUM58433.1	AKA62048.1
RNA-DNA and DNA-DNA helicase ATI	AZV01323.1	AUM58432.1	AKA62049.1
inhibitor of prohead protease	AZV01324.1	AUM58431.1	AKA62050.1
head outer capsid protein	AZV01325.1	AUM58430.1	AKA62051.1
hypothetical protein	AZV01326.1	AUM58429.1	AKA62052.1
RNA polymerase ADP-ribosylase	AZV01327.1	AUM58428.1	AKA62054.1
hypothetical protein	AZV01328.1	AUM58427.1	AKA62053.1
hypothetical protein	AZV01329.1	AUM58426.1	AKA62055.1
DNA ligase	AZV01330.1	AUM58424.1	AKA62057.1
hypothetical protein	AZV01331.1	AUM58423.1	AKA62058.1
hypothetical protein	AZV01332.1	AUM58422.1	AKA62059.1
hypothetical protein	AZV01333.1	AUM58421.1	AKA62060.1
hypothetical protein	AZV01335.1	AUM58419.1	AKA62061.1
hypothetical protein	AZV01336.1	AUM58418.1	AKA62063.1
rIII lysis inhibition accessory protein	AZV01337.1	AUM58416.1	AKA62065.1
head assembly cochaperone with Gr	AZV01338.1	AUM58415.1	AKA62066.1
tail fiber protein	AZV01339.1	AUM58414.1	AKA62067.1
hypothetical protein	AZV01340.1	AUM58413.1	AKA62068.1
hypothetical protein	AZV01341.1	AUM58411.1	AKA62070.1
phospho-2-dehydro-3-deoxyheptona	AZV01342.1	AUM58410.1	AKA62071.1
hypothetical protein	AZV01343.1	AUM58409.1	AKA62072.1
hypothetical protein	AZV01344.1	AUM58408.1	AKA62073.1
hypothetical protein	AZV01345.1	AUM58407.1	AKA62074.1
3'-phosphatase, 5'-polynucleotide kir	AZV01346.1	AUM58406.1	AKA62075.1
hypothetical protein	AZV01348.1	AUM58404.1	AKA62077.1
hypothetical protein	AZV01350.1	AUM58402.1	AKA62078.1
hypothetical protein	AZV01351.1	AUM58401.1	AKA62079.1
inhibitor of host transcription	AZV01352.1	AUM58400.1	AKA62080.1
RNA ligase 1 and tail fiber attachmer	AZV01353.1	AUM58399.1	AKA62081.1
endonuclease II	AZV01354.1	AUM58398.1	AKA62082.1
aerobic NDP reductase small subunit	AZV01355.1	AUM58397.1	AKA62083.1
ribonucleotide reductase of class Ia (	AZV01356.1	AUM58395.1	AKA62085.1
hypothetical protein	AZV01358.1	AUM58393.1	AKA62087.1
hypothetical protein	AZV01359.1	AUM58392.1	AKA62088.1
thymidylate synthase	AZV01360.1	AUM58389.1	AKA62089.1
hypothetical protein	AZV01361.1	AUM58388.1	AKA62090.1
hypothetical protein	AZV01362.1	AUM58387.1	AKA62091.1
hypothetical protein	AZV01363.1	AUM58386.1	AKA62092.1

dihydrofolate reductase	AZV01364.1	AUM58385.1	AKA62093.1
single-stranded DNA-binding protein	AZV01365.1	AUM58382.1	AKA62094.1
DNA helicase loader	AZV01366.1	AUM58381.1	AKA62095.1
late promoter transcription accessor	AZV01367.1	AUM58380.1	AKA62096.1
dsDNA binding protein	AZV01368.1	AUM58379.1	AKA62097.1
RNase H	AZV01369.1	AUM58378.1	AKA62098.1
long tail fiber proximal subunit	AZV01370.1	AUM58377.1	AKA62099.1
long tail fiber proximal connector	AZV01371.1	AUM58376.1	AKA62100.1
long tail fiber distal connector	AZV01372.1	AUM58375.1	AKA62101.1
long tail fiber distal subunit	AZV01373.1	AUM58374.1	AKA62102.1
distal long tail fiber assembly catalys	AZV01374.1	AUM58373.1	AKA62103.1
holin lysis mediator	AZV01375.1	AUM58372.1	AKA62104.1
hypothetical protein	AZV01376.1	AUM58371.1	AKA62105.1
hypothetical protein	AZV01377.1	AUM58370.1	AKA62106.1
hypothetical protein	AZV01378.1	AUM58369.1	AKA62107.1
hypothetical protein	AZV01379.1	AUM58368.1	AKA62108.1
anti-sigma 70 protein	AZV01380.1	AUM58367.1	AKA62109.1
hypothetical protein	AZV01381.1	AUM58366.1	AKA62110.1
transcriptional regulator	AZV01382.1	AUM58365.1	AKA62111.1
hypothetical protein	AZV01383.1	AUM58362.1	AKA62112.1
hypothetical protein	AZV01384.1	AUM58361.1	AKA62113.1
hypothetical protein	AZV01385.1	AUM58364.1	AKA62114.1
DNA topoisomerase	AZV01386.1	AUM58363.1	AKA62115.1
nucleoid disruption protein	AZV01387.1	AUM58360.1	AKA62116.1
DNA endonuclease IV	AZV01388.1	AUM58359.1	AKA62117.1

CoreGenes analysis of vB_SsoS_008

The number of homologs in each column is : 50
Total number of genes in genome 1 : 83
% in common, with respect to genome 1: 60.24
Total number of genes in genome 2 : 78
% in common, with respect to genome 2: 64.1
Total number of genes in genome 3 : 80
% in common, with respect to genome 3: 62.5
Total number of genes in genome 4 : 82
% in common, with respect to genome 4: 60.98

Function	vB_SsoS_008 (MK335533)	T1 (AY216660)	phi2457T (MH917278)	SH6 (KX828710)
hypothetical protein	QAX92008.1	AAP49998.1	AYP69390.1	APC44920.1
hypothetical protein	QAX92013.1	AAP49926.1	AYP69405.1	APC44911.1
hypothetical protein	QAX92015.1	AAP49929.1	AYP69374.1	APC44973.1
hypothetical protein	QAX92016.1	AAP49930.1	AYP69376.1	APC44984.1
hypothetical protein	QAX92017.1	AAP49931.1	AYP69424.1	APC44970.1
hypothetical protein	QAX92023.1	AAP49935.1	AYP69380.1	APC44930.1
kinase	QAX92024.1	AAP49936.1	AYP69371.1	APC44960.1
hypothetical protein	QAX92026.1	AAP49939.1	AYP69370.1	APC44910.1
hypothetical protein	QAX92027.1	AAP49940.1	AYP69410.1	APC44980.1
hypothetical protein	QAX92028.1	AAP49941.1	AYP69392.1	APC44950.1
hypothetical protein	QAX92029.1	AAP49944.1	AYP69423.1	APC44918.1
hypothetical protein	QAX92030.1	AAP49945.1	AYP69419.1	APC44962.1
terminase small subunit	QAX92031.1	AAP49947.1	AYP69372.1	APC44944.1
terminase large subunit	QAX92032.1	AAP49948.1	AYP69351.1	APC44953.1
portal protein	QAX92033.1	AAP49949.1	AYP69353.1	APC44942.1
minor capsid protein	QAX92035.1	AAP49999.1	AYP69362.1	APC44922.1
major head subunit precursor	QAX92038.1	AAP49950.1	AYP69355.1	APC44972.1
hypothetical protein	QAX92040.1	AAP49951.1	AYP69377.1	APC44955.1
hypothetical protein	QAX92041.1	AAP49952.1	AYP69361.1	APC44983.1
hypothetical protein	QAX92042.1	AAP49953.1	AYP69357.1	APC44931.1
hypothetical protein	QAX92043.1	AAP49954.1	AYP69397.1	APC44965.1
hypothetical protein	QAX92044.1	AAP49956.1	AYP69389.1	APC44986.1
hypothetical protein	QAX92045.1	AAP49957.1	AYP69381.1	APC44961.1
hypothetical protein	QAX92046.1	AAP49958.1	AYP69388.1	APC44925.1
hypothetical protein	QAX92047.1	AAP50000.1	AYP69399.1	APC44924.1
tail tape measure protein	QAX92052.1	AAP49961.1	AYP69348.1	APC44947.1
tail fibers protein	QAX92053.1	AAP49962.1	AYP69391.1	APC44974.1
minor tail protein	QAX92054.1	AAP49963.1	AYP69360.1	APC44909.1
minor tail protein	QAX92055.1	AAP49964.1	AYP69363.1	APC44919.1
tail assembly protein	QAX92056.1	AAP49965.1	AYP69369.1	APC44963.1
tail fiber protein	QAX92061.1	AAP49966.1	AYP69347.1	APC44985.1
exodeoxyribonuclease VIII	QAX92065.1	AAP49970.1	AYP69356.1	APC44928.1
recombination protein	QAX92066.1	AAP49971.1	AYP69367.1	APC44939.1
hypothetical protein	QAX92067.1	AAP49972.1	AYP69384.1	APC44921.1
tail fibers protein	QAX92069.1	AAP49973.1	AYP69350.1	APC44917.1
DNA primase/helicase	QAX92070.1	AAP49975.1	AYP69359.1	APC44959.1
hypothetical protein	QAX92072.1	AAP49976.1	AYP69379.1	APC44936.1
ATP-dependent helicase	QAX92073.1	AAP49977.1	AYP69349.1	APC44976.1
hypothetical protein	QAX92074.1	AAP49978.1	AYP69382.1	APC44943.1
DNA N-6-adenine-methyltransferase	QAX92075.1	AAP49979.1	AYP69364.1	APC44914.1
hypothetical protein	QAX92076.1	AAP49980.1	AYP69400.1	APC44946.1

hypothetical protein	QAX92077.1	AAP49981.1	AYP69411.1	APC44907.1
hypothetical protein	QAX92081.1	AAP49983.1	AYP69354.1	APC44977.1
hypothetical protein	QAX92082.1	AAP49985.1	AYP69420.1	APC44915.1
endolysin	QAX92083.1	AAP49986.1	AYP69408.1	APC44968.1
spanin	QAX92084.1	AAP49988.1	AYP69387.1	APC44951.1
hypothetical protein	QAX92087.1	AAP49989.1	AYP69386.1	APC44945.1
hypothetical protein	QAX92088.1	AAP49990.1	AYP69352.1	APC44969.1
hypothetical protein	QAX92089.1	AAP49992.1	AYP69383.1	APC44956.1
hypothetical protein	QAX92090.1	AAP49993.1	AYP69412.1	APC44952.1
